# The complex structure of GRL0617 and SARS-CoV-2 PLpro reveals a hot spot for antiviral drug discovery

**DOI:** 10.1038/s41467-020-20718-8

**Published:** 2021-01-20

**Authors:** Ziyang Fu, Bin Huang, Jinle Tang, Shuyan Liu, Ming Liu, Yuxin Ye, Zhihong Liu, Yuxian Xiong, Wenning Zhu, Dan Cao, Jihui Li, Xiaogang Niu, Huan Zhou, Yong Juan Zhao, Guoliang Zhang, Hao Huang

**Affiliations:** 1 State Key Laboratory of Chemical Oncogenomics, School of Chemical Biology and Biotechnology, Peking University Shenzhen Graduate School, Shenzhen, 518055 China; 2 Laboratory of Structural Biology and Drug Discovery, Peking University Shenzhen Graduate School, Shenzhen, 518055 China; 3 National Clinical Research Center for Infectious Diseases, Shenzhen Third People’s Hospital, Southern University of Science and Technology, Shenzhen, 518112 China; 4 College of Chemistry and Molecular Engineering, Beijing Nuclear Magnetic Resonance Center, Peking University, Beijing, 100871 China; 5 Shanghai Advanced Research Institute, Chinese Academy of Sciences, Shanghai, China

**Keywords:** Structural biology, Drug discovery, Nanocrystallography, Viral infection

## Abstract

SARS-CoV-2 is the pathogen responsible for the COVID-19 pandemic. The SARS-CoV-2 papain-like cysteine protease (PLpro) has been implicated in playing important roles in virus maturation, dysregulation of host inflammation, and antiviral immune responses. The multiple functions of PLpro render it a promising drug target. Therefore, we screened a library of approved drugs and also examined available inhibitors against PLpro. Inhibitor GRL0617 showed a promising in vitro IC_50_ of 2.1 μM and an effective antiviral inhibition in cell-based assays. The co-crystal structure of SARS-CoV-2 PLpro^C111S^ in complex with GRL0617 indicates that GRL0617 is a non-covalent inhibitor and it resides in the ubiquitin-specific proteases (USP) domain of PLpro. NMR data indicate that GRL0617 blocks the binding of ISG15 C-terminus to PLpro. Using truncated ISG15 mutants, we show that the C-terminus of ISG15 plays a dominant role in binding PLpro. Structural analysis reveals that the ISG15 C-terminus binding pocket in PLpro contributes a disproportionately large portion of binding energy, thus this pocket is a hot spot for antiviral drug discovery targeting PLpro.

## Abstract (web summary)

The SARS-CoV-2 papain-like protease (PLpro) is of interest as a drug target. Here, the authors identify GRL0617 as a PPI (protein–protein interaction) inhibitor of SARS-CoV-2 PLpro that inhibits its deISGylating activity and present the mechanism of action of the compound through the GRL0617-bound PLpro crystal structure and NMR studies.

## Introduction

The COVID-19 pandemic has caused devastating damage to the world and it has resulted in over 12 million confirmed cases and over half a million deaths as of July 14, 2020^1^. The novel SARS-CoV-2 coronavirus is the etiological agent responsible for the pandemic, and it belongs to the beta coronavirus family^2–4^. Similar to the two beta coronaviruses, SARS and MERS, which have caused pandemic or epidemic in human history, the novel SARS-CoV-2 also causes severe acute respiratory syndromes^5,6^. Unexpectedly, SARS-CoV-2 has been reported to have more mild symptoms but much higher transmission rate^7,8^, therefore it has caused the biggest catastrophe to the world healthcare since the Spanish flu in 1918–1920^9^. Four previously deemed promising antiviral drugs, i.e., Remdesivir, Hydroxychloroquine, Lopinavir, and Interferon, showed no or little effect on hospitalized COVID-19 patients, in WHO global solidarity clinical trials^10^. Encouragingly, the UK started rollout of an mRNA vaccine developed by Pfizer/BioNtech in early December 2020, with its long-term safety and efficacy to be assessed. Meanwhile, progress has been made in the discovery of antibodies for COVID-19. Therefore, anti-SARS-CoV-2 drugs are urgently needed.

As a positive strand RNA virus, SARS-CoV-2 encodes two functional proteases, i.e., the papain-like protease (PLpro) and the 3-chymotrypsin-like cysteine protease (Mpro or 3CLpro). The major function of Mpro is to cleave the viral polyproteins, which is critical for virus maturation, replication, and invasion. Several potent Mpro covalent inhibitors have been reported and their co-crystal structures provided potential opportunities for structure-based drug optimization^11–14^. SARS-CoV PLpro is a cysteine protease with multiple major functions, including processing of the viral polyprotein chain for viral protein maturation, dysregulating host inflammation responses through deubiquitylation, and impairing the host type I interferon antiviral immune responses by removing interferon stimulated gene 15 (ISG15) modifications^15–17^. ISG15 modification (ISGylation) is the covalent conjugation of ISG15 protein (MW = 17.1 kDa) to protein substrates and this process is known to inhibit virus replication^18–20^. SARS-CoV-2 PLpro (MW = 35.6 kDa), sharing ~83% sequence identity with SARS-CoV PLpro, contains an N-terminal ubiquitin-like (UBL) domain and a C-terminal ubiquitin-specific protease (USP) domain with implicated catalytic functions of cleaving ubiquitin (Ub) or ISG15 modifications from host proteins^21^.

Besides Mpro, PLpro has been considered another potentially promising target for drug discovery to treat COVID-19^22^. Due to the urgent need of therapies for the pandemic, the strategy of repurposing approved drugs or optimizing new compounds has been employed to fight COVID-19^14,23–25^. Accordingly, we describe our efforts in screening of a compound library of drugs approved by FDA or CFDA (China FDA) against SARS-CoV-2 PLpro, and structural characterization of the interactions between a promising drug lead and PLpro. The co-crystal structure of PLpro in complex with the compound GRL0617 and its antiviral effect provided direct proof of druggability of PLpro and the mechanism of action of the compound. Further structural and biophysical analysis reveals that the C-terminus of ISG15 plays a dominant role in its binding with PLpro through extensive hydrogen bonds and electrostatic interactions. Therefore, the ISG15-C-terminus binding cleft in PLpro is a hot spot for antiviral drug discovery.

## Results

### High-throughput screening of a library of approved drugs against PLpro

To repurpose existing drugs to inhibit the SARS-CoV-2 PLpro, we initiated screening of a 2040-compound library of drugs approved by FDA or CFDA against SARS-CoV-2 PLpro (Supplementary Table 1). First, we set up a FRET assay to characterize the enzymatic activity of SARS-CoV-2 PLpro based on an established assay for SARS-CoV PLpro^26^. The recombinant full-length SARS-CoV-2 PLpro protein was expressed in *Escherichia coli*, and subsequently purified using his-tag chromatography and size exclusion chromatography (Supplementary Fig. 1). A commercially available fluorogenic peptide substrate Arg-Leu-Arg-Gly-Gly-AMC (RLRGG-AMC), representing the C-terminal residues of ubiquitin, was used to report the enzymatic activity of PLpro. The first round of screening provided ~30 compounds with over 50% inhibition at 100 μM. Because the FDA approved drug Tioguanine (6-TG) has been previously tested on SARS-CoV and MERS PLpro proteins with effective inhibitions^27,28^, we determined its potency against SARS-CoV-2 PLpro and the IC_50_ value was 72 ± 12 μM, so we used it as a positive control throughout the screening. Hits from the first round of screening went into the second round of validation using the same enzymatic assay. After removing compounds with poor solubility, strong reactivity, or high intrinsic fluorescence, seven relatively potent compounds including 6-TG were measured for IC_50_. These seven drugs showed modest IC_50_ values ranging from 29 to 91 μM (Supplementary Fig. 3). Although these compounds can potentially provide a starting point for further optimization, their low potency implies a need of large amounts of resources and time input.

### Identification of GRL0617 as an inhibitor for SARS-CoV-2 PLpro

**Fig. 1:**
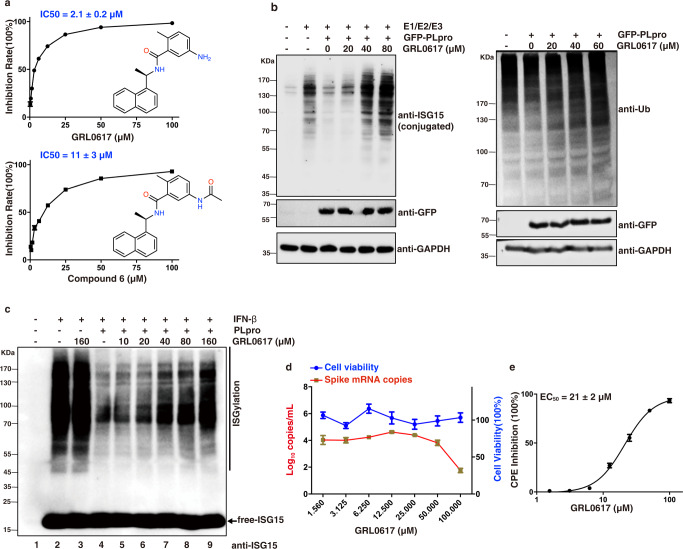
Inhibitory activity of GRL0617 against SARS-CoV-2 PLpro. **a** The inhibitory activity of GRL0617 and compound 6 against PLpro was measured using the peptide RLRGG-AMC as a substrate. IC_50_ was presented as mean ± SEM, *n* = 3 independent experiments. **b** In-cell deISGylating (left) and deubiquitinating (right) activities of PLpro, HEK293T cells were transfected for 24 h with plasmids encoding GFP-PLpro, ISG15, and E1(Ube1L)/E2(UbcH8)/E3(HECR5) enzymes, alone or in combination. Cells were treated for an additional 24 h with indicated concentrations of GRL0617. Cell lysates were subjected to immunoblotting with anti-ubiquitin, anti-ISG15, and anti-GFP antibodies. GAPDH served as a loading control. A representative from three independent experiments is shown. **c** HEK293T cells were treated with or without 500 U/mL interferon β (IFN-β) for 48 h. The cell extracts were incubated with purified recombinant PLpro (100 nM) and indicated concentrations of GRL0617 for 60 min at 37 °C, followed by immunoblotting analysis with anti-ISG15. A representative from three independent experiments is shown. **d** Antiviral activity of GRL0617 on SARS-CoV-2 and the cytotoxicity of GRL0617 on Vero E6 cells. Vero E6 cells were infected with SARS-CoV-2 using a multiplicity-of-infection (MOI) of 0.01. The quantification of absolute viral RNA copies (per mL) in the supernatant at 48 h post-infection was determined by qRT-PCR analysis. The cytotoxicity of GRL0617 on Vero E6 cells was measured using CCK8. All data are shown as mean ± SEM, *n* = 3 independent experiments. **e** EC_50_ was presented as mean ± SEM, *n* = 3 independent experiments, the cytopathic effect (CPE) analysis revealed an EC_50_ of 21 ± 2 μM.

Parallelly, we cherry-picked GRL0617 and its analog compound 6 from promising SARS-CoV PLpro inhibitors^26,29^ based on high sequence identity between the SARS-CoV and SARS-CoV-2 PLpro proteins (Supplementary Fig. 2). The in vitro IC_50_ values of GRL0617 and compound 6 against SARS-CoV-2 PLpro were 2.1 ± 0.2 μM and 11 ± 3 μM, respectively (Fig. 1a). The compound 6, as an acetamide derivative of GRL0617, did not show improved potency in the in vitro FRET assay. Our data suggested that GRL0617 is a promising lead compound and therefore it was subjected to further antiviral, structural, and mechanistic studies. Our identification of GRL0617 and its analogs along with their potencies are in line with recent studies by the Pegan group^30^, the Dikic group^31^, and the Komander group^32^.

### Inhibition of the in-cell deubiquitinating and deISGylating activity of PLpro by GRL0617

To assess whether GRL0617 can inhibit the *in cyto* deubiquitinating and deISGylating activity of SARS-CoV-2 PLpro, we transfected HEK293T cells with plasmids of PLpro and the ISGylation machinery (Ube1L, UbcH8, HECR5, and ISG15) and then treated with GRL0617 at different concentrations for 24 h. Our data (Fig. 1b) showed that the deubiquitinating activity of SARS-CoV-2 PLpro is weak but its deISGylating activity is relatively strong, which is consistent with recent publications^30–33^. SARS-CoV-2 PLpro is capable of reversing the ISGylation and polyubiquitination (to a much lesser extent) of cellular substrates (Fig. 1b). Furthermore, addition of GRL0617 caused inhibition of SARS-CoV-2 PLpro and resulted in partially recovered poly-ubiquitin-conjugates and ISG15-conjugates (Fig. 1b). Moreover, we further used interferon β (IFN-β) to induce the ISGylation in HEK293T cells which generated a remarkable amount of ISG15-conjugated cellular substrates (Fig. 1c, lanes 1 and 2). The addition of 160 μM of GRL0617 alone to cell lysate had no observable effect to ISGylation (Fig. 1c, lanes 2 and 3), which demonstrated the selectivity of the compound over other deISGylating enzymes in cells, such as USP18. Indeed, GRL0617 showed no inhibition effect on the recombinant mouse USP18 protein (Supplementary Fig. 5g), which is consistent with other studies^26,31^. Further addition of purified SARS-CoV-2 PLpro (100 nM) to cell lysate efficiently reduced ISG15-conjugated proteins (Fig. 1c, lanes 2 and 4). In contrast, addition of GRL0617 to cell lysate together with PLpro recovered the ISGylation in a dose-dependent manner (Fig. 1c, lanes 5–9) with significant band recovery seen at 40 μM of GRL0617. Clearly, GRL0617 inhibited the deISGylation activity of PLpro through an on-target effect.

### Antiviral activity of the inhibitor GRL0617

To further validate the potential of PLpro as an antiviral drug target, we tested GRL0617 for its inhibitory activity in Vero E6 cells infected with SARS-CoV-2 at a multiplicity-of-infection (MOI) of 0.01. The mRNA copy numbers of the viral spike protein were monitored to evaluate antiviral activity of the compound. Based on the dose-dependent response, GRL0617 showed a clear inhibition of viral replication and 100 μM of GRL0617 resulted in over 50% inhibition. No apparent cytotoxicity on Vero E6 cells was observed in our assay with concentrations up to 100 μM (Fig. 1d). The cytopathic effect (CPE) analysis revealed an EC_50_ of 21 ± 2 μM (Fig. 1e). This *in cyto* antiviral potency of GRL0617 is also in line with other recent studies^30,31^.

### The co-crystal structure of SARS-CoV-2 PLpro in complex with GRL0617

**Fig. 2:**
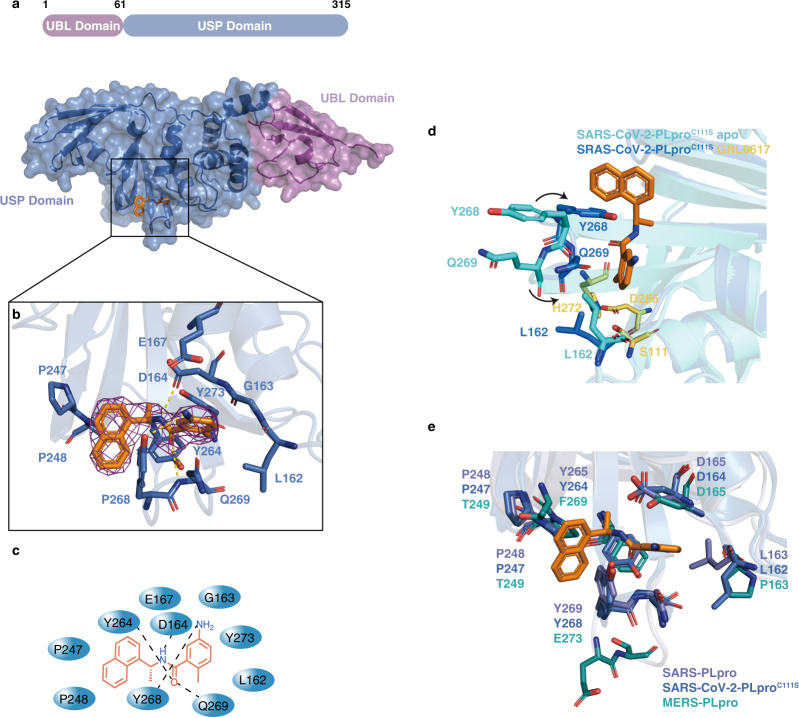
Structural and mechanistic analysis of SARS-CoV-2 PLpro^C111S^ in complex with GRL0617, and in comparison with SARS-CoV and MERS PLpro. **a** Surface and cartoon structures of SARS-CoV-2 PLpro in complex with GRL0617 (orange sticks) showing the N-terminal UBL domain (magenta) and C-terminal USP domain (marine). **b** The binding pocket of GRL0617 in PLpro. The PLpro residues involved in GRL0617 binding are shown as marine sticks. The 2Fo-Fc omit map (contour level = 1.6 σ, shown as purple mesh). **c** Schematic diagram of SARS-CoV-2 PLpro^C111S^/GRL0617 interactions shown in (**b**). **d** Comparison of GRL0617-bound (marine) PLpro^C111S^ and unbound (cyan) PLpro^C111S^ (PDB ID: 6WRH [10.2210/pdb6wrh/pdb]) structures. Y268 and Q269 on the BL2 loop shifted toward GRL0617 upon binding. GRL0617 shown as orange sticks; the catalytic triad residues (S111 in place of C111, H272, and D286) are shown in yellow; Y268 and Q269 are shown in marine in bound state, and in cyan in unbound state. **e** Comparisons of the binding sites of SARS-CoV PLpro/GRL0617 (slate sticks, PDB ID: 3E9S [10.2210/pdb3e9s/pdb])^26^, SARS-CoV-2 PLpro^C111S^/GRL0617 (marine sticks), and MERS-CoV PLpro (deep teal sticks, PDB ID: 4RNA [10.2210/pdb4rna/pdb])^37^.

A co-crystal structure would be crucial to understand the mechanism of inhibition of SARS-CoV-2 by GRL0617, therefore we set out to solve the co-crystal structure. However, the crystals of wild-type SARS-CoV-2 PLpro were difficult to grow. Therefore, we turned to grow co-crystals of SARS-CoV-2 PLpro^C111S^ in complex with GRL0617 by incubating the compound with the protein before setting up crystal trays. The obtained co-crystal diffracted at 3.2 Å (Fig. 2a–e and Supplementary Table 2). When our manuscript was in the review process, a few groups also reported the co-crystal structures of SARS-CoV-2 PLpro and GRL0617 or its analogs^34,35^. The crystal of PLpro/GRL0617 belongs to the space group I4_1_22 with one protein molecule in each asymmetric unit. SARS-CoV-2 PLpro has two domains, i.e., the N-terminal UBL domain and the C-terminal USP domain (Fig. 2a). Based on the B factor analysis, the palm and thumb regions in the USP domain have lower B factor values than the UBL domain and the fingers region of the USP domain, indicating that the palm and thumb regions are relatively more rigid than rest of the structure. As shown in the 2Fo-Fc electron density map (Fig. 2a, b) as well as in the difference Fo-Fc electron density map (Supplementary Fig. 4b), GRL0617 resides in a pocket in the palm region of PLpro. GRL0617 is apart from the catalytical triad (including S111 in place of C111, H272, and D286) of PLpro with a minimum distance of 7.5 Å to S111 (from the methyl of the 4-Methylbenzenamine moiety of GRL0617 to the sidechain oxygen of S111). Therefore, GRL0617 inhibits SARS-CoV-2 PLpro in a non-covalent manner. The GRL0617-bound PLpro structure is overall similar to the available apo-structure of PLpro^C111S^ (PDB 6WRH) with a backbone RMSD of 0.76 Å, except for two residues on the BL2 loop, i.e., Y268 and Q269 (Fig. 2d). Upon binding to GRL0617, the sidechains of Y268 and Q269 shifted toward GRL0617 to form polar and hydrophobic interactions with the compound and stabilized its binding (Fig. 2d). Specifically, the sidechain oxygen of Y268 forms a hydrogen bond with the amino group on the benzene ring of GRL0617, and another hydrogen bond of the backbone amino group of Q269 with the carbonyl oxygen of GRL0617 (Fig. 2b, c). In comparison with the apo-wild-type (wt-, PDB 6W9C [10.2210/pdb6w9c/pdb]) or C111S (PDB 6WRH [10.2210/pdb6wrh/pdb]) structures of SARS-CoV-2 PLpro, the BL2 loop in GRL0617-bound PLpro structure shifted toward the compound to form a T-shaped π-π stacking with the naphthalene group of GRL0617 and it also formed a deeper pocket to better accommodate the compound (Fig. 2e and Supplementary Fig. 5a, d, e). Other polar interactions include the hydrogen bonds between D164 and the amide NH of GRL0617, as well as between Y264 and the carbonyl oxygen of GRL0617. In addition, hydrophobic integration also contributed to the binding of GRL0617 to PLpro, e.g., the naphthalene group of GRL0617 is involved in the interactions with aromatic residues Y264 and Y268, and the hydrophobic sidechains of P247 and P248 (Fig. 2b, c).

Since GRL0617 is capable of inhibiting both SARS-CoV and SARS-CoV-2, it is of interest to understand its selectivity on coronaviral PLpro proteins from a structural biology perspective. It has been reported that naphthalene-based compounds have low to zero potency toward MERS PLpro^36,37^. The superposition of GRL0617 on a surface model of the MERS PLpro structure^37^ indicated that the original pocket in MERS PLpro might be too shallow to allow GRL0617 to bind with extensive contacts, and the naphthalene moiety of GRL0617 would also be in a steric clash with T249 of MERS PLpro (Supplementary Fig. 5c). In contrast to SARS and SARS-CoV-2, the BL-2 loop of MERS is one residue longer, but it lacks the critical Y268 of SARS-CoV-2 which played a critical role in encircling GRL0617 in the co-crystal structure (Supplementary Fig. 2). The extra residue of MERS PLpro may rearrange the hydrogen-bond interaction network of the BL2 loop and the lack of the aromatic tyrosine clearly resulted in the removal of the T-shaped π-π stacking and van der Waals interactions with the naphthalene group of GRL0617 (Fig. 2e and Supplementary Fig. 5a–e). Our enzymatic assay confirmed the lack of inhibition of MERS PLpro by GRL0617 or compound 6, which is consistent with our structural analysis (Supplementary Fig. 5f) and a recent study^31^.

### GRL0617 is a protein–protein interaction (PPI) inhibitor revealed by NMR

**Fig. 3:**
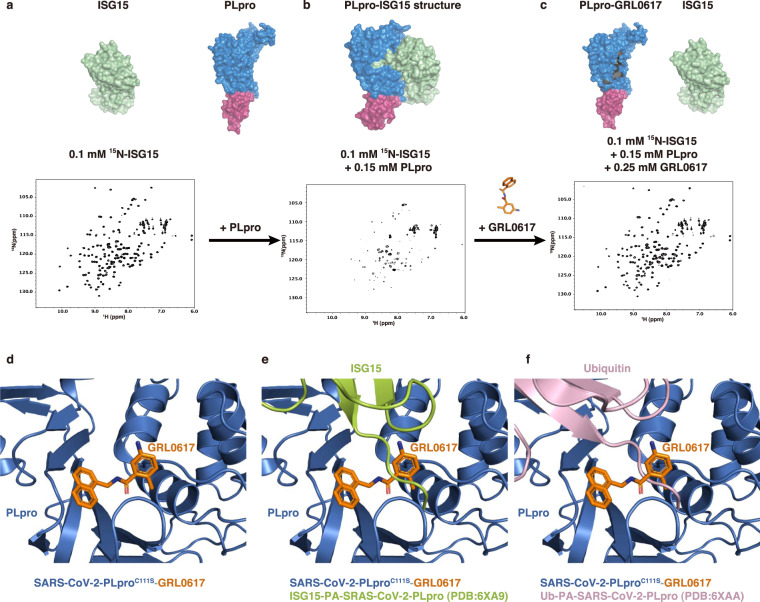
NMR studies show that GRL0617 blocks the binding of ISG15 to SARS-CoV-2 PLpro. **a** ^1^H,^15^N-HSQC spectrum of ^15^N-ISG15. **b** HSQC spectrum of ^15^N-ISG15 (0.1 mM) and 0.15 mM PLpro. Peak broadening and peak intensity loss indicate binding of ISG15 to PLpro. **c** HSQC spectrum of ^15^N-ISG15 (0.1 mM) in the mixture of 0.15 mM PLpro and 0.25 mM GRL0617. Recovery of peak intensity suggests that GRL0617 binds to PLpro and displaces ISG15. **d** SARS-CoV-2 PLpro^C111S^/GRL0617 structure in cartoon model. **e** ISG15 in the complex structure of human ISG15 C-UBL-PA/SARS-CoV-2 PLpro (PDB 6XA9 [10.2210/pdb6xa9/pdb]) was superimposed on (**d**) showing steric clash of GRL0617 with the C-terminal tail of ISG15. **f** Ub in the complex structure of UbPA/SARS-CoV-2 PLpro (PDB 6XAA [10.2210/pdb6xaa/pdb]) was superimposed on (**d**), showing steric clash of GRL0617 with the C-terminal tail of Ub.

Because GRL0617 is a non-covalent inhibitor binding in the USP domain, i.e., the catalytic domain of SARS-CoV-2 PLpro, it is of interest to see if GRL0617 would disrupt the interactions between ISG15 or Ub and PLpro. Solution state nuclear magnetic resonance (NMR) was employed to characterize the binding of ISG15 or Ub to PLpro as well as the perturbation of their bindings by GRL0617. 2-D NMR ^1^H,^15^N-HSQC spectrum of ^15^N-ISG15 (0.1 mM) showed typical features for a well-folded protein with well-dispersed cross peaks (Fig. 3a). The addition of 0.15 mM SARS-CoV-2 PLpro into 0.1 mM ^15^N-ISG15 caused drastic peak broadening and peak intensity loss, which is a characteristic of PPIs in the intermediate chemical exchange regime (Fig. 3b). Increasing concentrations of GRL0617 were added into the mix of 0.1 mM ^15^N-ISG15 and 0.15 mM SARS-CoV-2-PLpro, a dose-dependent response of peak intensity recovery was evident, which suggested that GRL0617 competes with ISG15 for the binding site in PLpro, and blocks the binding of ISG15 to PLpro (Supplementary Fig. 6a). The superposition of the HSQC spectra of 0.1 mM ^15^N-ISG15 only and the 0.1 mM ^15^N-ISG15/0.15 mM PLpro/0.25 mM GRL0617 mixture showed that these two spectra are essentially identical (Supplementary Fig. 6c), which indicated that GRL0617 is a potent binder to PLpro and almost completely abolished the binding of ISG15 to PLpro at a molar ratio of 1.67 (0.25 mM/0.15 mM). No peak shifting was observed in the superimposed HSQC (Supplementary Fig. 6b), suggesting that GRL0617 is a bona fide binder of PLpro rather than ISG15 because the HSQC spectrum of ^15^N-ISG15 is not disturbed at all by 2.5 excess molar ratio (0.25 mM/0.10 mM) of GRL0617.

In comparison with the available complex structure of SARS-CoV-2 PLpro with Ub^32^ (PDB ID: 6XAA [10.2210/pdb6xaa/pdb]) (Fig. 3f) or ISG15^31,32^ (PDB ID: 6XA9 [10.2210/pdb6xa9/pdb] and 6YVA [10.2210/pdb6yva/pdb]) (Fig. 3e), GRL0617 binds in the S1 site (for binding the C-terminal lobe of ISG15) and blocks the access of the C-terminal tail of proximal Ub or ISG15 to the active site of SARS-CoV-2 PLpro, respectively. Titrations of PLpro into ^15^N-Ub caused minimal peak shifting or peak broadening even at a high molar ratio of 3 (Supplementary Fig. 7), showing much weaker binding for PLpro with monoUb compared with ISG15. Consequently, GRL0617 was not further titrated into the ^15^N-Ub/PLpro mixture. Taken together, our NMR and X-ray analysis indicate that GRL0617 is a potent PPI inhibitor for PLpro by blocking the binding of ISG15 to PLpro.

### The C-terminus of ISG15 is dominant for ISG15/SARS-CoV-2 PLpro binding

As seen in Supplementary Fig. 6c, superimposed NMR spectra indicated an almost complete disruption of interactions of the N- and C-UBL domains of ISG15 with PLpro. It is intriguing that GRL0617 actually only blocked the binding of the C-terminal tail of ISG15, but it efficiently abolished the binding of both the N- and C-globular UBL domains of ISG15 with PLpro. Therefore, GRL0617, as a small-molecule compound (MW = 304.3), occupies a cleft near the active site and exerts a dominant negative effect for the rest of ISG15 binding to SARS-CoV-2 PLpro.

**Fig. 4:**
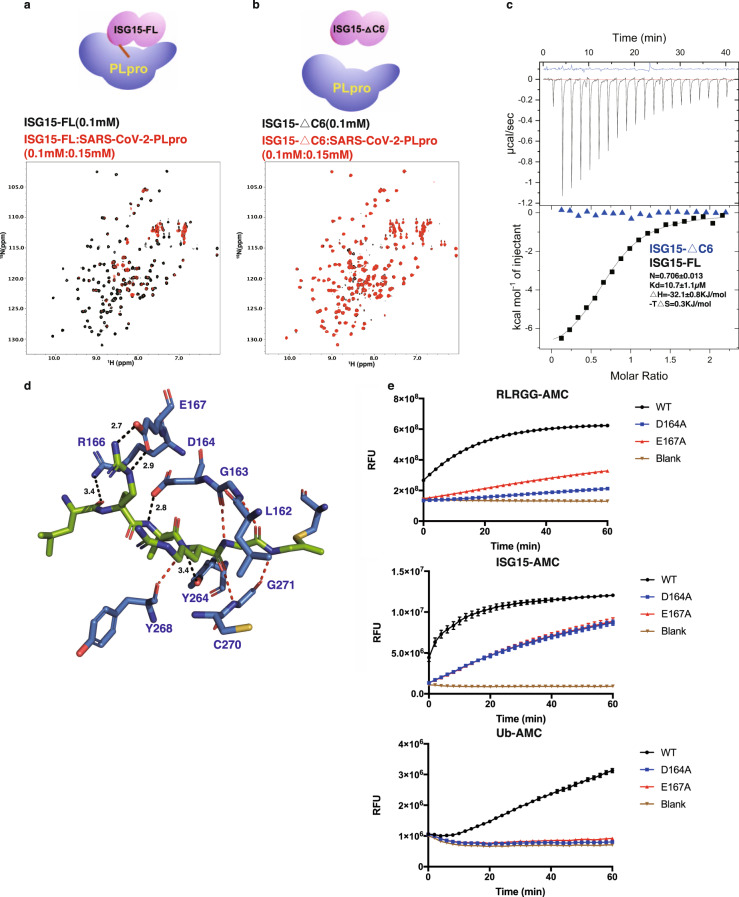
The C-terminal tail of ISG15 is dominant for binding SARS-CoV-2 PLpro. **a** Superposition of the ^1^H,^15^N-HSQC spectra of ^15^N-ISG15-FL (0.1 mM) and the mixture of ^15^N-ISG15 (0.1 mM)/0.15 mM SARS-CoV-2 PLpro. Massive peak broadening and peak intensity loss indicate binding of ISG15-FL to PLpro. **b** Superposition of the ^1^H,^15^N-HSQC spectra of ^15^N-ISG15-ΔC6 (0.1 mM) and the mixture of ^15^N-ISG15-ΔC6 (0.1 mM)/SARS-CoV-2 PLpro (0.15 mM). Negligible peak perturbation indicates minimum binding of ISG15-ΔC6 to PLpro. **c** ITC measurement for the binding of SARS-CoV-2 with ISG15-FL (black) and ISG15-ΔC6 (blue), respectively. **d** Structural analysis of the complex structure of ISG15/SARS-CoV-2 PLpro (PDB 6XA9 [10.2210/pdb6xa9/pdb]) shows that the sidechains (black dashed lines) of D164 and E167 of SARS-CoV-2 PLpro are involved in the binding with the C-terminal tail of ISG15, other interactions are involved with the backbone (red dashed lines). **e** DUB cleavage assay using Ub-AMC, ISG15-AMC, or peptide-AMC shows that D164A and E167A mutants have impaired enzyme activity compared with wild-type SARS-CoV-2 PLpro. Data are presented as mean ± SEM, *n* = 3 independent experiments.

To further confirm the important role of the C-terminal tail of ISG15, we generated a truncated construct ISG15-ΔC6 (removing the C-terminal LRLRGG). As shown in the superimposed ^1^H,^15^N-HSQC spectra of ^15^N-ISG15-FL and ^15^N-ISG15-ΔC6 (Supplementary Fig. 8), ISG15-ΔC6 has a fold similar to the full-length ISG15 (ISG15-FL). However, the superimposed ^1^H,^15^N-HSQC also indicates that the interactions between ISG15 and SARS-CoV-2 PLpro were abolished by removal of the C-terminus of ISG15, as minimal peak shifting or peak broadening were observed for ^15^N-ISG15-ΔC6/SARS-CoV-2 (molar ratio = 1:1.5) as compared with the massive peak broadening for ^15^N-ISG15-FL/SARS-CoV-2 (molar ratio = 1:1.5) (Fig. 4a, b). Isothermal titration calorimetry (ITC) experiments confirmed that removal of the C-terminus of ISG15 is detrimental to the binding of ISG15 and SARS-CoV-2 PLpro (Fig. 4c) (Table 1). Two complex structures of mouse full-length ISG15/SARS-CoV-2 PLpro (6VYA [10.2210/pdb6yva/pdb]) and human ISG15 C-UBL-PA/SARS-CoV-2 PLpro (PDB 6XA9 [10.2210/pdb6xa9/pdb]) were recently solved by the Dikic group^31^ and the Komander group^32^, respectively. Further structural analysis of the human ISG15 C-UBL-PA/SARS-CoV-2 PLpro reveals that the PLpro backbone of L162, G163, Y268, C270, and G271 as well as sidechains of D164, R166, E167, and Y264 were involved in the interactions with the C-terminus of ISG15 through hydrogen bonds and electrostatic interactions. (Fig. 4d). Two mutants D164A and E167A were subsequently generated, and their enzymatic activities of cleaving Ub tags or ISG15 tags were examined using Ub-AMC or ISG15-AMC, respectively. Both D164A and E167A showed impaired activity on Ub and ISG15 cleaving (Fig. 4e). The cleavage of peptide substrate RLRGG-AMC also indicated diminished enzymatic activities for these mutants. These two mutants did not show observable activity on Ub-AMC at 1 μM substrate concentration, presumably because SARS-CoV-2 PLpro cleaves Ub-AMC almost 10-fold less efficiently than ISG15-AMC^30^.

**Table 1:** The binding affinities of ISG15 and truncated mutants with SARS-CoV-2/SARS/MERS PLpro.

	SARS-CoV-2-PLpro	SARS-PLpro	MERS-PLpro
ISG15-FL	10.7 ± 1.1	20.50 ± 4.48^64^	59.3 ± 12.7^64^
ISG15-△C6	N.D.*	N.D.*	N.D.*
ISG15-△C5	N.D.*	—	—
ISG15-△C4	N.D.*	—	—

*N.D., not detected; —, not performed.

We also examined the binding of ISG15-ΔC6 with SARS-CoV or MERS PLpro using ^1^H,^15^N-HSQC spectra. Similar to SARS-CoV-2 PLpro, the other two orthologs also showed minimum interactions (Supplementary Fig. 9a–c). ITC results confirmed lack of interactions between ISG15-ΔC6 and SARS-CoV-2, SARS-CoV, or MERS PLpro (Supplementary Fig. 9d–f). Another two truncated ISG15 mutants, i.e., ISG15-ΔC5 (removing C-terminal RLRGG) and ISG15-ΔC4 (removing C-terminal LRGG) were also generated and tested for their bindings with SARS-CoV-2 PLpro by ITC (Table 1 and Supplementary Fig. 9g, h). Similar to the shorter construct ISG15-ΔC6, ISG15-ΔC5 and ISG15-ΔC4 showed no observable binding with SARS-CoV-2 PLpro. These binding results suggest that the C-terminus of ISG15 is dominant for its binding with SARS-CoV-2 PLpro.

### The extensive interaction network between ISG15 C-terminus and SARS-CoV-2 PLpro revealed by complex structures

Previous studies reveal that the C-terminal UBL domain of ISG15 is sufficient for binding with MERS PLpro^38^ or mammal USP18^39^. Based on a complex structure of full-length ISG15 and MERS PLpro, it was suggested that C-terminal tail of ISG15 plays an important role in the hydrogen-bond network formed by ISG15 and MERS PLpro^40^. Other studies also suggested that the C-terminal RLRGG of Ub is responsible for a major part of interaction between Ub and SARS-CoV^16^, MERS PLpro^16,41^, or human DUBs^42^.

**Fig. 5:**
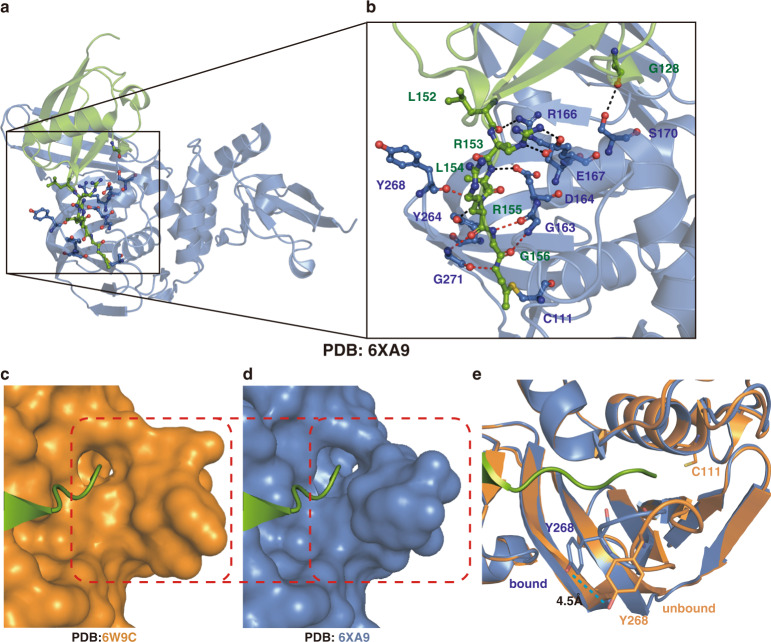
The extensive hydrogen-bond and electrostatic interaction network between ISG15 C-terminus and SARS-CoV-2 PLpro. **a** The complex structure of human ISG15 C-UBL domain and SARS-CoV-2 PLpro (PDB 6XA9 [https://doi.org/10.2210/pdb6xa9/pdb]). **b** Close-up view for interactions of the C-terminus of ISG15 (green) with PLpro (marine), sidechain interactions in black dashed lines, and backbone interactions in red dashed lines. **c** Surface model showing the superposition of the C-terminus of ISG15 on apo-PLpro (PDB 6W9C [10.2210/pdb6w9c/pdb]). **d** Surface model showing the C-terminus of ISG15 in PLpro (PDB 6XA9 [10.2210/pdb6xa9/pdb]). **e** Cartoon model showing the shift of BL2 loop from the apo-state (orange) to bound state (marine), a distance of 4.5 Å was labeled for the shifting of sidechain η-OH of Y268 in PLpro.

Accordingly, we analyzed available human C-UBL-ISG15-PA/SARS-CoV-2 PLpro and mouse ISG15-FL/SARS-CoV-2 PLpro complex structures using the PISA (proteins, interfaces, structures, and assemblies) program^43^ (Fig. 5a, b and Supplementary Fig. 10). Comparing these two structures, it is clear that the majority of the interactions between ISG15 and PLpro is from the C-UBL domain of ISG15. In two recent studies^31,32^, the PLpro residues V66, F69, Y171, and N156 were reported for interacting with the N- or C-globular domains of ISG15^31,32^. In addition, intermolecular interactions with the C-terminus of ISG15 involve PLpro residues G163, D164, E167, R166, Y264, Y268, and G271 (Fig. 5a, b and Supplementary Fig. 10). The backbone of G163, Y268, G271 and the sidechains of D164, R166, E167, Y264 formed hydrogen bonds and electrostatic interactions with PLpro (Fig. 5b and Supplementary Fig. 10c). By comparing the structures of apo- and ISG15-bound SARS-CoV-2 PLpro, a shift of the BL2 loop of PLpro was observed (Fig. 5c–e). In apo-SARS-CoV-2 PLpro, the ISG15-C-terminus binding pocket takes an open conformation. Upon binding to ISG15, the PLpro BL2 loop shifts toward the substrate, i.e., the C-terminus of ISG15, and firmly holds the C-terminus through a hydrogen-bond and electrostatic interaction network (Fig. 5). Using the sidechain OH of Y268 as a reference, the BL2 loop shifted 4.5 Å to encircle the C-terminus of ISG15 (Fig. 5e), which also happened to the binding of GRL0617 in the same pocket (Fig. 2d).

## Discussion

Our biochemical, structural, and antiviral data of SARS-CoV-2 PLpro support the claim that PLpro is a promising drug target for COVID-19 treatment. Our co-crystal structure of PLpro^C111S^ in complex with the potent inhibitor GRL0617 and its antiviral effect on Vero 6 cells validated that SARS-CoV-2 PLpro is a druggable target for SARS-CoV-2. In two recent studies, it was reported that the inhibition of deISGylating activity of PLpro is linked to the antiviral activity of PLpro inhibitors^31,44^. Our studies provided the mechanism of action for GRL0617. GRL0617 blocks the binding of ISG15 or Ub to PLpro, naturally it will also inhibit the processing of viral polyproteins of SARS-CoV-2 since these viral polyproteins share similar substrate cleavage site with Ub and ISG15.

The dominant role of the C-terminus of ISG15 in binding SARS-CoV-2 PLpro is intriguing. ISG15 contains N- and C-terminal globular domains and a short C-terminal tail (residues RLRGG), which is often considered part of the C-domain^45^. Recent structural analysis reported that the SARS-CoV-2 PLpro S1 site is mainly for high ISG15 activity while the S2 site determines substrate selectivity^31,32^. In our study, we show that the short and linear C-terminal tail of ISG15 dominates its binding with PLpro. Because the tail is short and flexible, it offers transient and reversible interactions with its binding partner, i.e., PLpro in this case, which ensures optimal enzyme efficiency and high enzymatic turnover. Since the C-terminus of Ub is also heavily involved in its interactions with MERS or SARS-CoV PLpro^16,41^, primarily interacting with the C-terminus of Ub or ISG15 could be a general strategy for viral DUBs to attack host immune system.

**Fig. 6:**
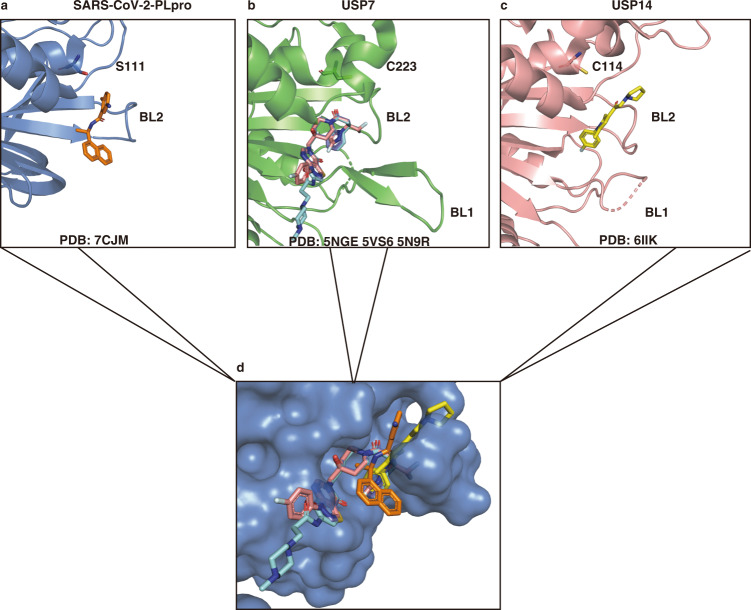
GRL0617 occupies the same binding pocket in the USP domain as other USP7 and USP14 inhibitors. **a** Binding pocket of GRL0617 (orange) in SARS-CoV-2 PLpro^C111S^ mutant (PDB 7CJM [10.2210/pdb7cjm/pdb]), the S111 was labeled in place of C111. **b** Binding pocket of FT671 (PDB 5NGE [10.2210/pdb5gne/pdb], pink), XL188 (PDB 5VS6 [10.2210/pdb5vs6/pdb], cyan), and ALM2 (PDB 5N9R [10.2210/pdb5n9r/pdb], purple) in USP7 with active Cys223 label. **c** Binding pocket of IU1 (yellow) in USP14 (PDB 6IIK [10.2210/pdb6iik/pdb]) with active Cys114 label; the BL1 and BL2 loops were labeled in (**a**, **b**, **c**). **d** Superposition of GRL0617, USP7, and USP14 in the ISG15 C-terminus binding pocket in SARS-CoV-2 PLpro.

The ISG15 C-terminus binding cleft in PLpro contributes a disproportionately large portion of the binding energy compared with the rest of the protein, therefore this is a hot spot pocket for antiviral PPI drug discovery. Small-molecule drugs often occupy hot spots on PPI interfaces and inhibit target proteins^46^. Because PLpro is a viral DUB with Ub and ISG15 cleavage functions, we compared our co-crystal structure with the structures of known USP7 and USP14 inhibitors to see if they share the same binding sites. Indeed, several non-covalent USP7 or USP14 inhibitors occupy the same pocket in the USP domain (Fig. 6) and block the binding of the C-terminus of Ub^47–49^. The binding site of USP7 inhibitors FT671 (PDB 5NGE [10.2210/pdb5nge/pdb])^47^, XL188 (PDB 5VS6 [10.2210/pdb5vs6/pdb])^48^, and ALM2 (PDB 5N9R [10.2210/pdb5n9r/pdb])^49^ largely overlap in the Ub-C-terminus binding cleft near the active Cys223; the USP14 inhibitor IU1 (PDB 6IIK [10.2210/pdb6iik/pdb])^50^ and GRL0617 reside in the same pocket encircled by the BL2 loop (Fig. 6a, c). The superposition of all these DUB inhibitors on PLpro (Fig. 6d), shows that they are all at the same Ub or ISG15 C-terminus binding cleft, illustrating arguably the most important pocket in SARS-CoV-2 PLpro for the discovery of PPI inhibitors. In addition, analysis of available co-crystal structures of inhibitor-bound DUBs suggests that stabilization of the inactive conformations of DUB^51^, and/or structural plasticity of the BL1/BL2 loops in DUB are also potential mechanisms of inhibition^52^. Since several independent high-throughput screening assays targeting SARS-CoV-2 using approved drugs have been unsuccessful^32,53^, structure-based drug discovery for this hot spot in PLpro and optimization of GRL0617 or its analogs with PLpro would be a promising approach for combating COVID-19. Recently reported covalent peptidic inhibitors targeting the active C111 also bind in this pocket in PLpro^33^.

Although the seven approved drugs obtained in our screening show low potency against PLpro, we cannot rule out the potentials of these drugs to therapeutically treat COVID-19 because they may have higher antiviral activities through other more complex mechanisms, e.g., the 6-TG^44^.

In summary, we report the co-crystal structure of SARS-CoV-2 PLpro and GRL0617. We also found that the C-terminus of ISG15 is dominant in its binding with PLpro. The ISG15 C-terminus binding cleft in PLpro is a hot spot for non-covalent PPI inhibitor discovery. Our study implicates that it may be an efficient approach to focus on this pocket for future efforts of drug discovery targeting SARS-CoV-2 PLpro.

## Methods

### Plasmids construction, protein expression, and purification

Protein sequence for SARS-CoV-2 PLpro (amino acids, 746-1060) of Nsp3 protein from SARS-CoV-2 (Nsp3; YP_009742610.1), protein sequence for SARS PLpro (amino acids, 723-1037) of Nsp3 protein from SARS (Nsp3; NP_828862.2), and protein sequence for MERS PLpro (amino acids, 627-950) of Nsp3 protein from MERS(Nsp3; YP_009047231.1) were codon-optimized (Supplementary Table 3), synthesized, and subcloned into pET28a vector with N-terminal His-tag and TEV protease site (General Biosystems, China). The C111S, D164A, and E167A mutations were introduced into the SARS-CoV-2-PLpro with a QuikChange site-directed mutagenesis kit (Agilent Technologies, USA) and primers (Supplementary Table 4). Protein sequence for *Homo sapiens* ISG15 (amino acids, 1-157) (ISG15; NP_005092.1) was synthesized and subcloned into pET28a with a tandem N-terminal His-tag, SUMO-tag, and TEV-protease site. ISG15 truncated mutants in the C-terminus (ΔC6, ΔC5, ΔC4) were constructed from above pET28a-SUMO-ISG15 plasmid by PCR and homologous recombination technology. Human Ub was codon-optimized, synthesized, and subcloned into pET28a with an N-terminal His-tag and TEV-protease site. Protein sequence for Mus musculus USP18 (amino acids, 46-368) (USP18; CAJ18436.1P) was codon-optimized, synthesized, and subcloned into pBac vector with tandem N-terminal His-tag, SUMO-tag, and TEV protease site (Genewiz, China). All plasmids were verified by DNA sequencing analysis.

The plasmids were subsequently transformed into *E. coli* BL21 (DE3) cells. Protein expression was carried out in LB medium. *E. coli* cells were grown in LB medium at 37 °C until OD_600_ reaches 0.8–1.0, 0.5 mM Isopropyl β-D-1-thiogalactopyranoside (IPTG) and 100 mM ZnSO_4_ were added, and cells were growing overnight at 18 °C. For the production of ^15^N-labeled ISG15 and Ub, protein samples were prepared by growing bacteria in M9 medium containing ^15^NH_4_Cl.

Cell pellets were resuspended in buffer A (30 mM Tris, 400 mM NaCl, 30 mM imidazole, 2 mM β-ME, pH 8.5) with the addition of 1 mM phenylmethylsulfonyl fluoride (PMSF) and lysed using sonication. Cell lysate was subsequently centrifuged at 40,000*g* at 4 °C for 1 h. The supernatant was further loaded on a Ni-NTA column and then purified using buffer B (30 mM Tris, 400 mM NaCl, 300 mM imidazole, 2 mM β-Me, pH 8.5) on an AKTA Pure purification system (GE Healthcare). A second step of purification was carried out on a Superdex 200 gel filtration column using Buffer C (30 mM Tris, 100 mM NaCl, 1 mM DTT, pH 8.5, for crystallization) or Buffer D (30 mM Tris, 100 mM NaCl, 1 mM DTT, pH 7.4, for measuring the enzymatic activities and NMR tests). The fractions were pooled and concentrated to 10 mg/mL and stored at −80 °C.

The mUSP18 was prepared with the MultiBac system^54,55^ as described previously^56^. It is briefly described as follows: pBac-His-SUMO-TEV-mUSP18 was transformed into DH10EmBacY cells and the positive clones were screened and selected with a blue-white screening protocol. The virus bacmid was transfected into Sf9 cells (Thermo Fisher Scientific) with lipofectamine 2000 (Thermo Fisher Scientific). Sf9 cells were grown in Sf-900™ II SFM media (Thermo Fisher Scientific). The initial virus was harvested and successful infection was monitored by the measurement of yellow fluorescent protein expression using a fluorescence spectrophotometer. Western blotting was used to test the expression of mUSP18. For large-scale expression of mUSP18, 100 mL of Sf9 cells at a density of 1 × 10^6^ cells/mL were infected with 200–600 μL of the virus. The cells were kept at the same density until proliferation arrest. Then the cells were harvested for purification. The purification process is the same as for PLpro proteins.

### Cell culture and plasmid transfection

HEK293T(ATCC) and Vero E6 (Shanghai Institutes for Biological Sciences, Chinese Academy of Sciences) cells were cultured in DMEM (Dulbecco’s Modified Eagle Medium; Gibco) medium supplemented with 10% (vol/vol) FBS (Fetal Bovine Serum; Gibco) and penicillin (100 U/mL)/streptomycin (100 μg/mL). In transient-transfection experiments, cells at 70% confluency were transfected with plasmid DNA constructs using Lipofectamine 3000 reagents (Invitrogen, L3000015).

### Immunoblotting for detection of ISGylation

HEK293T cells were co-transfected with plasmids encoding Myc-tagged ISG15, pcDNA3,1-UBE1L (E1), UBCH8 (E2), or 3XFlag-HERC5 (E3) for 24 h. Cell extracts were prepared in RIPA lysis buffer (20 mM Tris-HCl (pH 7.5), 150 mM NaCl, 1 mM EDTA, 1% NP-40, 1% SDS, and protease inhibitor cocktail (Roche Diagnostics, Germany, 5892791001), pH 7.5. Protein concentrations were determined by Bradford assay (Bio-Rad Laboratories, USA, 500-0205). Equal amounts proteins (40 μg) were subjected to SDS-PAGE, followed by transferring to nitrocellulose membranes. Membranes were blocked with TBST containing 5% skim-milk for 1 h at room temperature and then overnight with ISG15 (1:1000, Thermo Fisher Scientific, USA, 703131), GFP (1:1000, Abclonal, China, AE012), GAPDH (1:5000, Abclonal, China, AC002), and Ubiquitin (1:1000, Cell Signal Technology, USA, #3936) antibodies at 4 °C. After three washing steps, membranes were incubated with HRP-conjugated secondary antibodies (1:10000, Santa Cruz Biotechnology, TX, sc-516102, 1:10000, Enzo, ADI-SAB-300) for 1 h at room temperature and visualized using ChemiDoc system (Bio-Rad Laboratories, USA).

In the assay of IFN-β (Sino Biological Inc., 10704-HNAS) induced ISGylation and the inhibition of PLpro by GRL0617, HEK293T cells were first treated with 500 U/mL IFN-β for 48 h and cell extracts were prepared in RIPA lysis buffer; 30 μg of extracts were mixed with purified recombinant PLpro (100 nM) and indicated concentrations of GRL0617 in the reaction buffer (50 mM Tris-HCl, 50 mM NaCl, 5 mM DTT, pH 7.5). Reactions were incubated at 37 °C for 1 h and prepared for immunoblotting analysis as indicated.

### PLpro activity assays and IC_50_ determination

In this study, PLpro activity was monitored using the substrate peptide-AMC (Z-Arg-Leu-Arg-Gly-Gly-AMC, Cat. No. 4027158, Bachem Bioscience). Experiments were performed in 384-well black non-binding plates (Cat. No. 3575, Corning) with a final reaction volume of 50 μL. The assay buffer contained 50 mM HEPES, pH 7.4, 0.01% Triton X-100 (v/v), 0.1 mg/mL BSA, and 2 mM DTT. PLpro was added to the plates at a final concentration of 100 nM. Enzyme reactions were initiated with 5 μL of peptide-AMC (final 50 μM) dissolved in the above assay buffer. Upon addition of peptide substrate, the fluorescence signals were monitored at 340 nm (excitation) and 450 nm (emission) with 3 min intervals in a 2104 EnVision Multilabel Plate Reader (PerkinElmer).

An approved drug library (TargetMol, USA), of 2040 compounds including drugs approved by US FDA and CFDA, was used. The first round screening reaction mixture included 100 nM PLpro, 50 μM substrate, and 100 μM compounds. The top 1.5% compounds of each plate were selected with a minimum 50% inhibition using the reaction in DMSO as a control. Around 30 compounds were tested in the second round of screening using the same assay and 23 compounds were excluded due to their reactivities, insolubility, or fluorescence interference. To determine the IC_50_ values of the remaining 7 compounds, a series of 8-point, 1 : 2 serial dilutions was performed from a highest starting concentration of 200 μM. Seven drugs used in this study were purchased from TargetMol (USA). The data were fitted using GraphPad Prism.

To test whether GRL0617 can inhibit mouse deISGylating enzyme USP18, its activity was assayed in the presence of an inhibitor with 250 nM ISG15-AMC (Boston Biochem) as substrate (excitation: 340 nm; emission: 450 nm).

The activities of wild-type PLpro, D164A, and E167A (all at 100 nM) were tested on peptide-AMC (100 μM), Ub-AMC (1 μM, Boston Biochem), or ISG15-AMC (1 μM) and were determined and compared.

### Crystallization and data collection

The complex of GRL0617 (Cat. No. HY-117043, MCE) with PLpro^C111S^ was crystallized by vapor diffusion in a sitting-drop format after a 20 h incubation of 9.5 mg/mL PLpro in the buffer (50 mM Tris, pH 8.5,100 mM NaCl) with 2 mM inhibitor at 4 °C. Immediately before crystallization, the sample was clarified by centrifugation. A 0.75 μL volume of the enzyme-inhibitor solution was then mixed with an equal volume of well solution containing 5 mM Cobalt (II) chloride hexahydrate; 5 mM Cadmium chloride hemi (pentahydrate); 5 mM Magnesium chloride hexahydrate; 5 mM Nickel (II) chloride hexahydrate; 0.1 M HEPES, pH 7.5; and 12% PEG 3350 and equilibrated against well solution at 12 °C. Before data collection, crystals were soaked in a cryo solution containing well solution, 400 μM inhibitor, and 20% glycerol. Crystals were flash-frozen in liquid N_2_. All diffraction data were collected at an in-house light source Rigaku MicroMax-007 HF and indexed, integrated, and scaled using CrysAlisPro and XDS^57^.

### Structure determination and data deposition

Structures were solved by molecular replacement using the apo-PLpro^C111S^ structure (PDB 6WRH [10.2210/pdb6wrh/pdb]) as the template. The MOLREP^58^ in CCP4^59^ was used for MR. Structures were refined in PHINEX^60^ with manual model building in Coot^61^. Detailed statistics on the data collection and the final models for crystallographic analysis are shown in Supplementary Table 2. Structural models were assessed using MolProbity^62^. Structural alignments and graphical representations were generated using PyMOL^63^.

### Isothermal titration calorimetry of ISG15 and its truncated mutants with PLpro proteins

ITC was performed using a Microcal-ITC200. One injection with 0.4 μL and subsequent 19 injections of 2 μL were performed at 25 °C with a reference power of 5 μcal/s. The ISG15 and its truncated mutants with SARS-CoV-2/SARS-CoV/MERS PLpro proteins were all in PBS buffer. For binding experiment of SARS-CoV-2 PLpro with ISG15 or ISG15-△C6, 92 μM PLpro was placed in the cell with 0.95 mM of ISG15 or 1 mM ISG15-△C6 in the syringe. For binding experiment of SARS-CoV or MERS PLpro with ISG15-△C6, 100 μM of SARS-CoV or MERS PLpro was placed in the cell with 1.2 mM or 1 mM ISG15-△C6 in the syringe. For binding experiment of SARS-CoV-2 PLpro with ISG15 truncated mutants (△C5, △C4), 100 μM of SARS-CoV-2 PLpro was placed in cell with 1–2 mM various ISG15 mutants in the syringe. The data were processed using Microcal-ITC200 analysis Software.

### Antiviral and cytotoxicity assay

For the assay, 1 × 10^4^ Vero E6 cells were seeded in triplicates in 96-well plates. After 20–24 h, fresh medium with different concentrations (100, 50, 25, 12.5, 6.25, 3.125, and 1.56 μM) of inhibitors, DMSO as a control, was replaced. For measuring the cytotoxicity, cells were incubated for 48 h followed by the cell viability test with CCK8 reagent. For measuring the antiviral activity, cells were kept in the medium with inhibitors for 1 h, followed by infection with SARS-CoV-2 virus strain BetaCoV/Shenzhen/SZTH-003/2020, which was clinically isolated from local patients, at MOI = 0.01. After a 2 h incubation, the virus–compound mixture was subsequently removed, and fresh medium containing candidate compounds (100, 50, 25, 12.5, 6.25, 3.125, and 1.56 μM) or DMSO was added, and cell growth was continued for 48 h. The viral RNA was extracted from the supernatant medium and the qRT-PCR assay was performed. The linearized plasmid containing the Spike gene of the SARS-CoV-2 virus was transcribed in vitro and was used to prepare a standard curve to quantify the copy number of the virus as previously described^11^. Briefly, the SARS-CoV-2 Spike gene were synthetized in pcDNA3.1 (Sangon, China). This vector was then linearized by single locus restriction endonuclease and subjected to in vitro transcription. The concentration of resulting RNA transcripts was measured by NanoDrop 2000. Microgram concentration of the plasmid can be transformed to the RNA copies. The first dilution is 10^7^ copies, then diluted to 10^2^ copies (6 diluted concentrations: 10^7^, 10^6^,10^5^,10^4^,10^3^,10^2^). The qRT-PCR assay was performed with the above template and the probe, and primers of Spike gene. Standard curves were prepared according to the results of PCR amplification. The standard curve formula is *y* = −3.2998x + 37.758, *R*^2^ = 0.9997. Primer and probe information: TaqMan primers for COVID-19 virus: 5′TCCTGGTGATTCTTCTTCAGG-3′ and 5′-TCTGAGAGAGGGTCAAGTGC-3′ and COVID-19 virus probe 5′-FAM-AGCTGCAGCACCAGCTGTCCA-BHQ1-3′. Data analysis was done with GraphPad Prism.

SARS-CoV-2-induced CPE on Vero E6 cells was observed and analyzed using reverse-phase light microscope. The inhibition effect of GRL0617 against SARS-CoV-2-induced cytopathogenic effect was measured under different concentrations. The cytopathic effect was examined for 3 days post-infection. The complete absence of cytopathic effect in an individual culture well was defined as protection. The value of EC_50_ was calculated using GraphPad prism software.

### NMR spectroscopy

NMR data were acquired at 25 °C on a 600 MHz Bruker AVANCE III spectrometer. The 600 MHz spectrometer was equipped with a 5 mm TCI Cryoprobe. In NMR titrations, the samples of 0.1 mM ^15^N-labeled ISG15 were incubated in the presence or absence of 0.15 mM PLpro with or without the indicated concentration of GRL0617 (0.05 mM, 0.15 mM, and 0.25 mM) were investigated in assay buffer containing 30 mM Tris, 100 mM NaCl, pH 7.4, 5% DMSO, and 10% D_2_O. For Ub titrations, the samples of 0.1 mM ^15^N-labeled Ub were incubated with PLpro (0.1 mM, 0.2 mM, and 0.3 mM). ^1^H,^15^N-HSQC titration spectra were collected for all the samples. All of the NMR spectra were processed using NMRPipe/NMRDraw and further analyzed using NMRView.

### Reporting summary

Further information on research design is available in the Nature Research Reporting Summary linked to this article.

## Supplementary information

Supplementary Information

Reporting Summary

## Data Availability

Coordinates and structure factors were deposited in the PDB under accession code 7CJM (GRL0617-bound PLproC111S). Protein sequence for SARS-CoV-2 PLpro (amino acids, 746-1060) of Nsp3 protein from SARS-CoV-2 (Nsp3; YP_009742610.1). Protein sequence for SARS PLpro (amino acids, 723-1037) of Nsp3 protein from SARS (Nsp3; NP_828862.2). Protein sequence for MERS PLpro (amino acids, 627-950) of Nsp3 protein from MERS (Nsp3; YP_009047231.1). Protein sequence for Homo sapiens ISG15 (amino acids, 1-157) (ISG15; NP_005092.1). Protein sequence for Mus musculus USP18(amino acids, 46-368) (USP18; CAJ18436.1 P). All relevant data are available upon request from the corresponding authors. Source data are provided with this paper.

## Source data

Source Data
