# Supplementary material for: The complex structure of GRL0617 and SARS-CoV-2 PLpro reveals a hot spot for antiviral drug discovery
Source: Nat Commun. 2021 Jan 20;12:488. doi: 10.1038/s41467-020-20718-8 (PMC7817691; doi:10.1038/s41467-020-20718-8)
Supplement: Supplementary file 1 — Supplementary Information [file 41467_2020_20718_MOESM1_ESM.pdf]

The Complex Structure of GRL0617 and SARS-CoV-2 PLpro Reveals a Hot Spot For Antiviral Drug Discovery

Authors: Ziyang Fu[1,2]†, Bin Huang[1,2]†, Jinle Tang[1,2]†, Shuyan Liu[3]†, Ming Liu[1,2], Yuxin Ye[1,2], Zhihong Liu[1,2], Yuxian Xiong[1,2], Wenning Zhu[1,2], Dan Cao[1,2], Jihui Li[1,2], Xiaogang Niu[4], Huan Zhou[5], Yong Juan Zhao[1], Guoliang Zhang[3]*, Hao Huang[1,2]*

[1]State Key Laboratory of Chemical Oncogenomics, School of Chemical Biology and Biotechnology, Peking University Shenzhen Graduate School, Shenzhen 518055, China;

[2]Laboratory of Structural Biology and Drug Discovery, Peking University Shenzhen Graduate School, Shenzhen 518055, China;

[3]National Clinical Research Center for Infectious Diseases, Shenzhen Third People's Hospital, Southern University of Science and Technology, Shenzhen 518112, China

[4]College of Chemistry and Molecular Engineering, Beijing Nuclear Magnetic Resonance Center, Peking University, Beijing 100871, China;

[5]Shanghai Advanced Research Institute, Chinese Academy of Sciences, Shanghai, China;

†These authors contributed equally to this work.

*To whom correspondence should be addressed:

Dr. Hao Huang, Tel: +86-0755-2603-2321; E-mail: huang.hao@pku.edu.cn

Dr. Guoliang Zhang, E-mail: szdsyy@aliyun.com

Supplementary Table 1: Small molecule screening data

Category	Parameter	Description
Assay	Type of assay	In *vitro* enzyme fluorescence-based activity assay (FRET)
	Target	SARS-CoV-2 PLpro
	Primary measurement	PLpro hydrolyzes the AMC labeled substrate (Peptide, RLRGG) and liberates fluorescent signal.
	Key reagents	Z-Arg-Leu-Arg-Gly-Gly-AMC, Cat. No. 4027158, Bachem Bioscience
	Assay Protocol	Experiments were performed in 384-well black non-binding plates (Cat. No. 3575, Corning) with a final reaction volume of 50 μL. The assay buffer contained 50 mM HEPES, pH 7.4, 0.01% Triton X-100 (v/v), 0.1 mg/ml BSA, 2 mM DTT. PLpro was added to the plates at a final concentration of 100 nM. Enzyme reactions were initiated with 5 μL of peptide-AMC (final 50 μM) dissolved in the above assay buffer. Upon addition of peptide substrate, the fluorescence signals were monitored at 340 nm (excitation) and 450 nm (emission).
	Additional comments	None
Library	Library size	2040 approved drugs at 10 mM in DMSO
	Library composition	Small molecules
	Source	TargetMol.
	Additional comments	None
Screen	Format	Black 384-well non-binding plate (Cat. No. 3575, Corning).
	Concentration(s) tested	100 μM, final DMSO concentration 1%.
	Plate controls	DMSO and 6-TG
	Reagent/compound dispensing system	with Eppendorf multiple channel pipette manually.
	Detection instrument and software	2104 EnVision Multilabel Plate Reader (PerkinElmer);Software: Envision workstation v.1.12.
	Assay validation/QC	Inhibition Rate > 50% with DMSO treatment. The average S/B was greater than 2.
	Correction factors	None

	Normalization	DMSO Control
	Additional comments	None
Post-HTS analysis	Hit criteria	Inhibition Rate > 50%
	Hit Rate	1.47%
	Additional assay(s)	Dose response
	Confirmation of hit purity and structure	Hit re-tested and then purchased as dry powders
	Additional comments	None

Supplementary Table 2: Data collection and refinement statistics

	PLpro+GRL0617
**Data collection**	
Space group	$I4_122$
Cell dimensions	
a, *b*, *c* (Å)	112.29 112.29 220.38
α, β, γ (°)	90 90 90
Resolution (Å)	22.14 - 3.2 (3.3-3.2)*
R_{sym} or R_{merge}	0.1902 (0.4874)
$I / \sigma I$	12.92 (4.27)
Completeness (%)	99.05 (99.83)
Redundancy	6.3 (6.6)
**Refinement**	
Resolution (Å)	22.14-3.2
No. reflections	11920
R_{work} / R_{free}	0.268 / 0.286
No. atoms	2533
Protein	2507
Ligand/ion	26
Water	-
B-factors	
Protein	41.08
Ligand/ion	24.52
Water	-
R.m.s. deviations	
Bond lengths (Å)	0.006
Bond angles (°)	0.86

*Values in parentheses are for highest-resolution shell

Supplementary Table 3: Sequences of codon optimized genes

Gene name	Gene sequence
**SARS-CoV-2-PLpro**	GAAGTTCGTACCATTAAGGTTTTTACCACCGTGGATAATATTAATCTGCA<br>TACCCAGGTTGTTGATATGAGCATGACCTATGGCCAGCAGTTTGGCCCG<br>ACCTATCTGGATGGTGCAGATGTGACCAAAATTAAGCCGCATAATAGCC<br>ATGAAGGCAAAACCTTTTATGTTCTGCCGAATGATGATACCCTGCGTGTT<br>GAAGCCTTTGAATATTATCATACCACCGATCCGAGTTTTCTGGGTCGTTA<br>TATGAGTGCCCTGAATCATACCAAAAAATGGAAATATCCGCAGGTGAAT<br>GGCCTGACCAGTATTAAGTGGGCAGATAATAATTGTTACCTGGCCACCG<br>CACTGCTGACCCTGCAGCAGATTGAACTGAAATTCAATCCGCCGGCACT<br>GCAGGATGCCTATTATCGCGCACGTGCCGGTGAAGCAGCCAATTTTTGC<br>GCACTGATTCTGGCCTATTGTAATAAGACCGTTGGTGAACTGGGCGATG<br>TGCGCGAAACCATGAGTTATCTGTTTCAGCATGCAAATCTGGATAGTTGT<br>AAACGTGTGCTGAATGTGGTGTGCAAAACCTGCGGTCAGCAGCAGACCA<br>CCCTGAAAGGCGTTGAAGCCGTGATGTATATGGGTACCCTGAGCTATGA<br>ACAGTTTAAAAAAGGTGTGCAGATTCCGTGTACCTGCGGCAAACAGGCA<br>ACCAAATATCTGGTTCAGCAGGAAAGCCCGTTTGTGATGATGAGTGCCC<br>CGCCGGCCCAGTATGAACTGAAACATGGTACCTTTACCTGCGCAAGCGA<br>ATATACCGGTAATTATCAGTGTGGTCATTATAAACATATCACCAGCAAA<br>GAAACCCTGTATTGTATTGATGGCGCCCTGCTGACCAAAAGCAGCGAAT<br>ATAAAGGTCCGATTACCGATGTGTTTTATAAAGAAAATAGCTACACCAC<br>CACCATTAAGTAA
**SARS-PLpro**	GAAGTTAAAACCATTAAGGTTTTTACCACCGTTGATAATACCAATCTGC<br>ATACCCAGCTGGTGGATATGAGCATGACCTATGGTCAGCAGTTTGGTCC<br>GACCTATCTGGATGGCGCAGATGTGACCAAAATTAAGCCGCATGTTAAT<br>CATGAAGGCAAAACCTTTTTCGTTCTGCCGAGCGATGATACCCTGCGCA<br>GCGAAGCATTTGAATATTATCATACCCTGGATGAAAGCTTTCTGGGTCGT<br>TATATGAGTGCACTGAATCATACCAAAAAATGGAAATTTCCGCAGGTTG<br>GTGGCCTGACCAGTATTAAGTGGGCAGATAATAATTGCTATCTGAGTAG<br>TGTTCTGCTGGCACTGCAGCAGCTGGAAGTTAAATTCAATGCACCGGCC<br>CTGCAGGAAGCATATTATCGCGCCCGTGCCGGTGACGCCGCCAATTTTT<br>GCGCCCTGATTCTGGCATATAGCAATAAGACCGTTGGCGAACTGGGTGA<br>CGTGCGTGAAACCATGACCCATCTGCTGCAGCATGCCAATCTGGAAAGT<br>GCAAAACGCGTTCTGAATGTTGTGTGTAAACATTGTGGTCAGAAAACCA<br>CCACCCTGACCGGTGTTGAAGCCGTGATGTATATGGGTACCCTGAGTTA<br>TGATAATCTGAAAACCGGCGTGAGCATTCCGTGTGTGTGTGGTCGCGAT<br>GCAACCCAGTATCTGGTTCAGCAGGAAAGTAGCTTTGTTATGATGAGTG<br>CACCGCCGGCAGAATATAAACTGCAGCAGGGCACCTTTCTGTGTGCCAA<br>TGAATATACCGGCAATTATCAGTGCGGCCATTATACCCATATTACCGCC<br>AAAGAAACCCTGTATCGCATTGATGGCGCCCATCTGACCAAAATGAGTG<br>AATATAAAGGTCCGGTTACCGATGTGTTTTATAAAGAAACCAGTTATAC<br>CACCACCATTAAGTAA
**MERS-PLpro**	ACCCAGCAGCTGACCATTGAAGTGCTGGTTACCGTTGATGGCGTGAATT<br>TTCGTACCGTGGTTCTGAATAATAAGAATACCTATCGCAGTCAGCTGGG<br>TTGTGTTTTCTTTAATGGTGCCGATATTAGTGATACCATTCCGGATGAAA<br>AACAGAATGGCCATAGCCTGTATCTGGCAGATAATCTGACCGCCGATGA<br>AACCAAAGCACTGAAAGAACTGTATGGTCCGGTGGACCCTACCTTTCTG<br>CATCGCTTTTATAGCCTGAAAGCCGCCGTGCATGGCTGGAAAATGGTTG<br>TGTGTGATAAAGTGCGTAGTCTGAAACTGAGTGATAATAATTGCTATCT

	GAACGCCGTGATTATGACCCTGGATCTGCTGAAAGATATTAAGTTTGTT<br>ATCCCGGCACTGCAGCATGCCTTTATGAAACATAAAGGCGGCGATAGCA<br>CCGATTTTATTGCACTGATTATGGCATACGGTAATTGCACCTTTGGTGCA<br>CCGGATGATGCAAGTCGTCTGCTGCATACCGTTCTGGCCAAAGCCGAAC<br>TGTGCTGCAGTGCCCGTATGGTTTGGCGCGAATGGTGCAATGTGTGCGG<br>CATTAAGGATGTGGTTCTGCAGGGTCTGAAAGCCTGTTGTTATGTGGGT<br>GTTCAGACCGTGGAAGATCTGCGCGCACGCATGACCTATGTTTGCCAGT<br>GTGGTGGCGAACGCCATCGTCAGCTGGTTGAACATACCACCCCGTGGCT<br>GCTGCTGAGTGGCACCCCGAATGAAAAACTGGTGACCACCAGTACCGCC<br>CCGGATTTTGTGGCCTTTAATGTGTTTCAGGGCATTGAAACCGCAGTGGG<br>TCATTATGTGCATGCCCGCCTGAAAGGTGGTCTGATTCTGAAATTTGATA<br>GTGGTACCGTTAGTAAAACCAGCGATTGGAAATGCAAAGTGACCGATGT<br>GCTGTTTCCGGGTCAGAAATATAGTAGTGATTGCAATTAA
**mUSP18**	GATAGCCCGCATGGTCTGGTTGGTTTACACAATATTGGTCAGACTTGTTG<br>TTTAAACTCTTTACTGCAAGTTTTTATGATGAACATGGACTTTCGCATGA<br>TTCTGAAACGTATCACCGTTCCGCGCAGCGCCGAAGAACGCAAACGTAG<br>CGTTCCGTTCCAGCTGCTGCTGTTACTGGAAAAGATGCAAGATAGTCGC<br>CAGAAGGCCGTTTTACCGACCGAACTGGTGCAATGTCTGCAGAAATACA<br>ACGTTCCGCTGTTCGTTCAGCATGATGCCGCACAGCTGTATTTAACCATC<br>TGGAATTTAACAAAGGACCAGATCACCGACACAGATTTAACCGAACGTT<br>TACAAGGTTTATTCACCATCTGGACCCAAGAATCTTTAATCTGTGTTGGT<br>TGCACCGCAGAGAGCAGCCGTCGCAGCAAGCTGCTGACTTTAAGTCTGC<br>CGTTATTCGACAAGGATGCAAAGCCGCTGAAGACTTTAGAGGACGCACT<br>GCGCTGTTTTGTGCAGCCGAAAGAGCTGGCAAGCAGCGACATGTGCTGC<br>GAAAGCTGTGGTGAGAAAACCCCGTGGAAACAAGTTCTGAAACTGACC<br>CATTTACCGCAAACTTTAACAATTCATTTAATGCGCTTTAGCGCCCGTAA<br>TAGCCGTACCGAAAAGATTTGTCATAGTGTGAACTTTCCGCAGTCTTTAG<br>ACTTTAGCCAAGTTCTGCCGACTGAAGAAGATCTGGGTGATACCAAGGA<br>GCAGAGCGAGATTCATTACGAGCTGTTCGCCGTGATCGCACATGTGGGC<br>ATGGCCGATTTCGGTCATTACTGCGCCTACATTCGCAATCCGGTGGATG<br>GCAAGTGGTTTTGCTTCAACGACAGCCACGTGTGCTGGGTGACTTGGAA<br>AGACGTGCAGTGTACATACGGCAACCATCGTTACCGTTGGCGTGAAACC<br>GCCTATTTACTGGTTTATACCAAAACTGGTAGC

Supplementary Table 4: Primers used in the article

Primer name	Primer sequence
SARS-CoV-2-PLpro-C111S-F	GCAGATAATAATTCTTACCTGGCCACCG
SARS-CoV-2-PLpro-C111S-B	CGGTGGCCAGGTAAGAATTATTATCTGC
SARS-CoV-2-PLpro-D164A-F	GTGAACTGGGCGCTGTGCGCGAAA
SARS-CoV-2-PLpro-D164A-B	TTTCGCGCACAGCGCCCAGTTCAC
SARS-CoV-2-PLpro-E167-F	GGCGATGTGCGCGCAACCATGAGTTAT
SARS-CoV-2-PLpro-E167-B	ATAACTCATGGTTGCGCGCACATCGCC
ISG15-△C6-F	GCACCGTGTTCATGAATTAACTCGAGCACCACCACCACC
ISG15-△C6-B	ATTCATGAACACGGTGCTCAGGG
ISG15-△C5-F	CATGAATCTGTAACGCCTGCGGGGAGGCTAAC
ISG15-△C5-B	GTTAGCCTCCCCGCAGGCGTTACAGATTCATG
ISG15-△C4-F	CATGAATCTGCGCTAACTGCGGGGAGGCTAAC
ISG15-△C4-B	GTTAGCCTCCCCGCAGTTAGCGCAGATTCATG

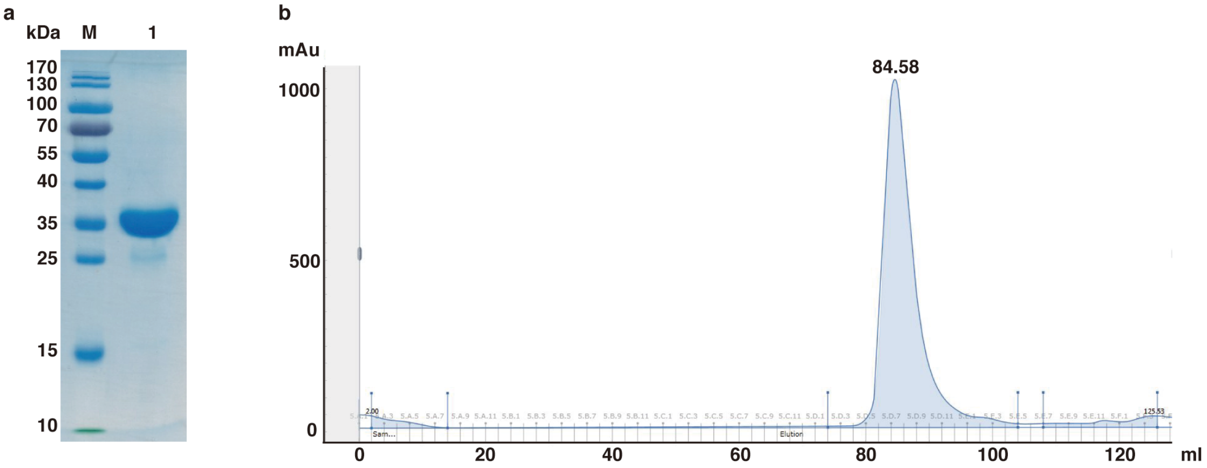

Supplementary Figure 1: The purification of recombinant SARS-CoV-2 PLpro protein.

a PLpro was expressed in *E. Coli* and further purified using His-tag chromatography and size-exclusion chromatography (SEC). Purified SARS-CoV-2 PLpro was stained using coomassie brilliant blue. A representative from three independent experiments is shown. **b** The peak at 84.58 mL on an S200 column corresponds to a monomer state of PLpro (MW = 35.6 kDa).

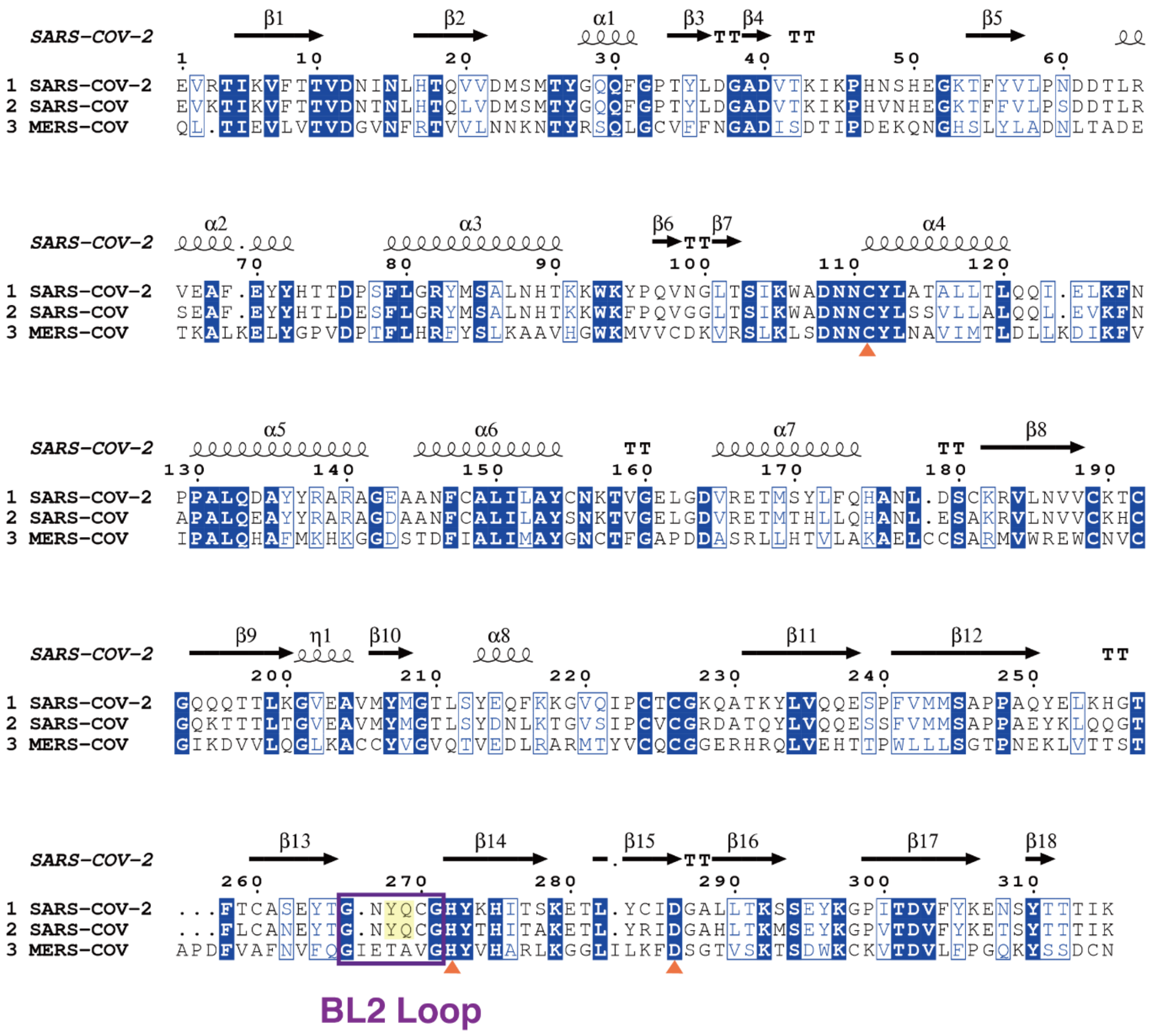

Supplementary Figure 2: Sequence alignment of SARS-CoV-2/SARS-CoV/MERS PLpro. Sequence alignment generated with MUSCLE/ESPRIPT[1,2] aligning PLpro sequences from SARS-CoV-2, SARS-CoV and MERS. Sequence numbering and secondary structure elements are shown according to the apo-structure of SARS-CoV-2 PLpro^{C111S} (PDB ID: 6W9C [10.2210/pdb6w9c/pdb] chain A). The catalytic triad residues are labeled with red trigonometry; the BL2 loop is shown with purple frame; two residues on the BL2 loop which has structural rearrangement upon GRL0617 binding are colored in yellow. α = α-helix, β = β-strand.

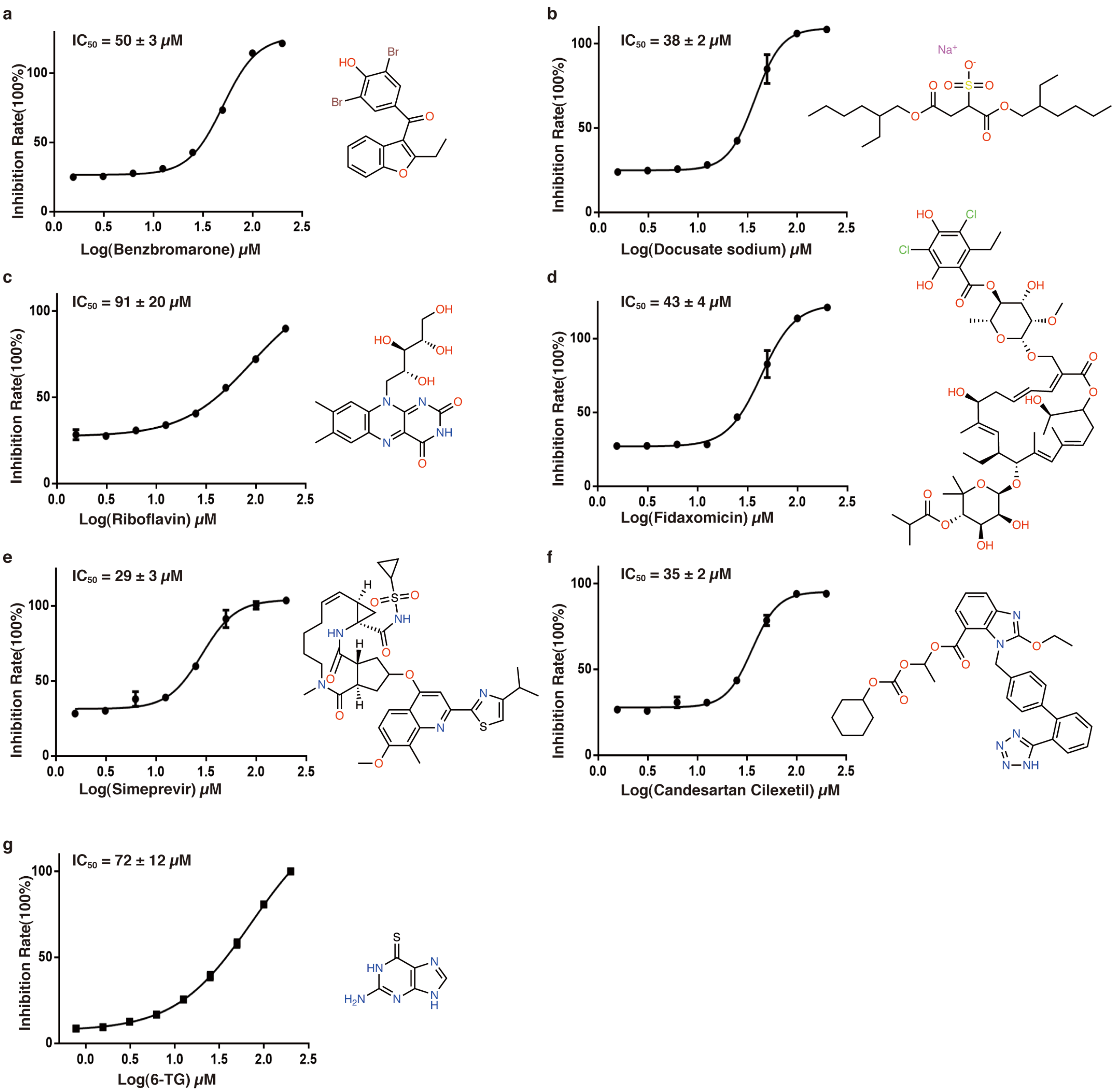

Supplementary Figure 3: The inhibitory activity of HTS hits against SARS-CoV-2 PLpro.

a-g Dose-response inhibition profile of drugs for half-maximum inhibitory concentration (IC_{50}) values were fitted using Graphpad prism. Data are presented as the means ± SEM, n = 3, independent experiments.

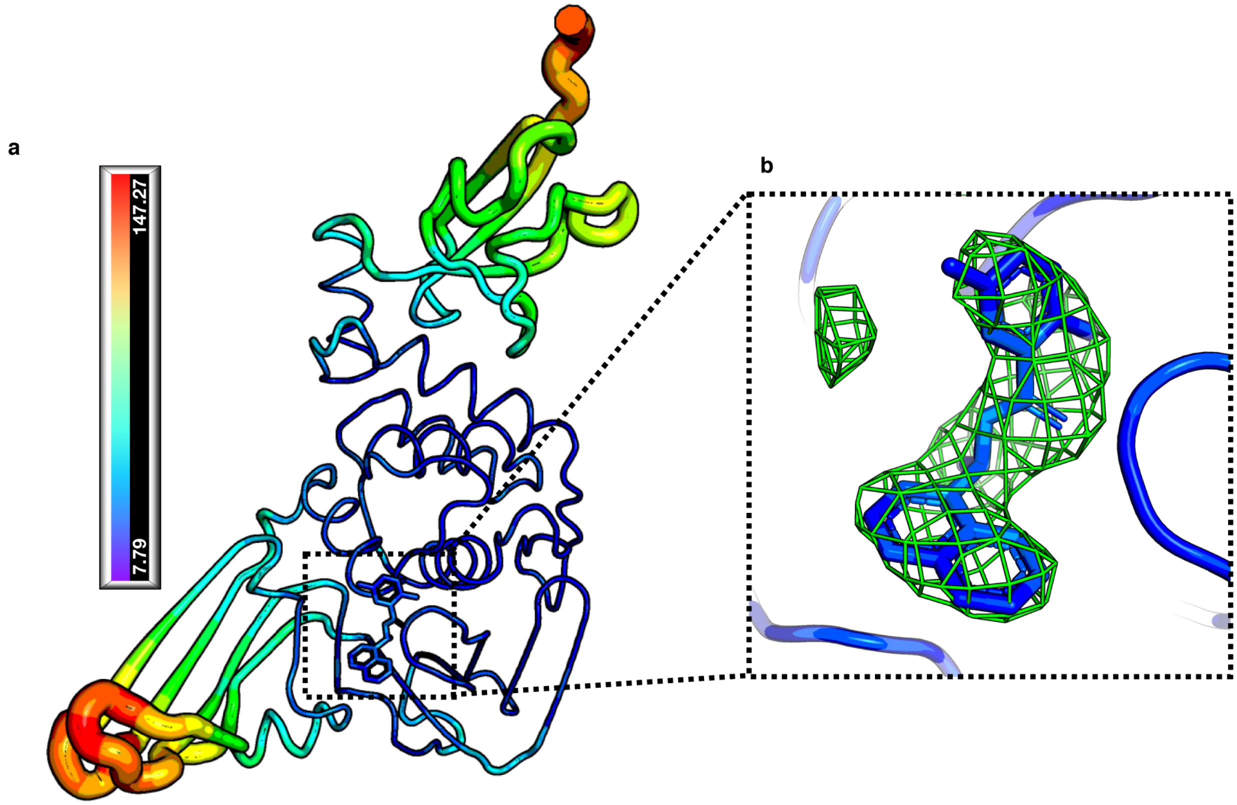

Supplementary Figure 4: Overall B factor of the complex structure and the compound Fo-Fc electron density map.

a The structure of PLpro-GRL0617^{C111S} is shown in B-factor scale where the UBL domain and the finger region of USP domain have higher B-factor values than the palm region of the USP domain, which is the binding site for GRL0617. **b** The close-up view of GRL0617 with the Fo-Fc electron density maps colored in green and contoured at 2.5 σ.

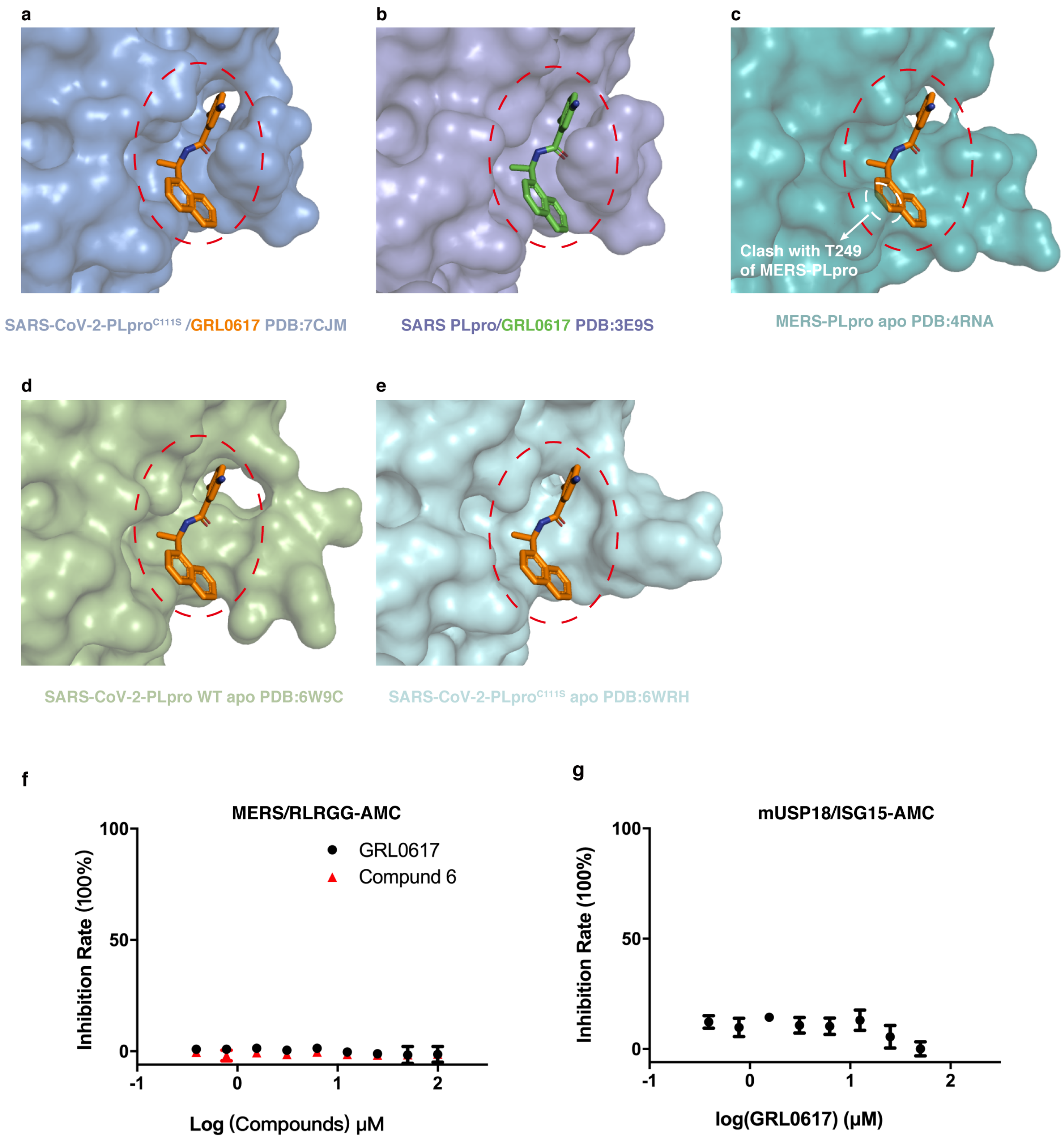

Supplementary Figure 5: Surface models of the GRL0617-Binding site in PLpro and the corresponding region in known viral PLpro crystal structures.

a SARS-CoV-2 PLpro^{C111S}/GRL0617. **b** SARS-CoV PLpro in complex with GRL0617 (PDB ID: 3E9S[10.2210/pdb3e9s/pdb]). **c** superposition of GRL0617 on apo- MERS PLpro (PDB ID: 4RNA[10.2210/pdb4rna/pdb]). **d** superposition of GRL0617 on apo-SARS-CoV-2 (PDB ID:

6W9C[10.2210/pdb6w9c/pdb]). **e** superposition of GRL0617 on apo-SARS-CoV-2 $PLpro^{C111S}$ (PDB ID: 6WRH[10.2210/pdb6wrh/pdb]). **f** GRL0617 and Compound 6 can't inhibit MERS PLpro using peptide-AMC as substrate. Data are presented as the means ± SD, n = 3 independent experiments. **g** GRL0617 can't inhibit mUSP18 using ISG15-AMC as substrate. Data are presented as the means ± SD, n = 3 independent experiments.

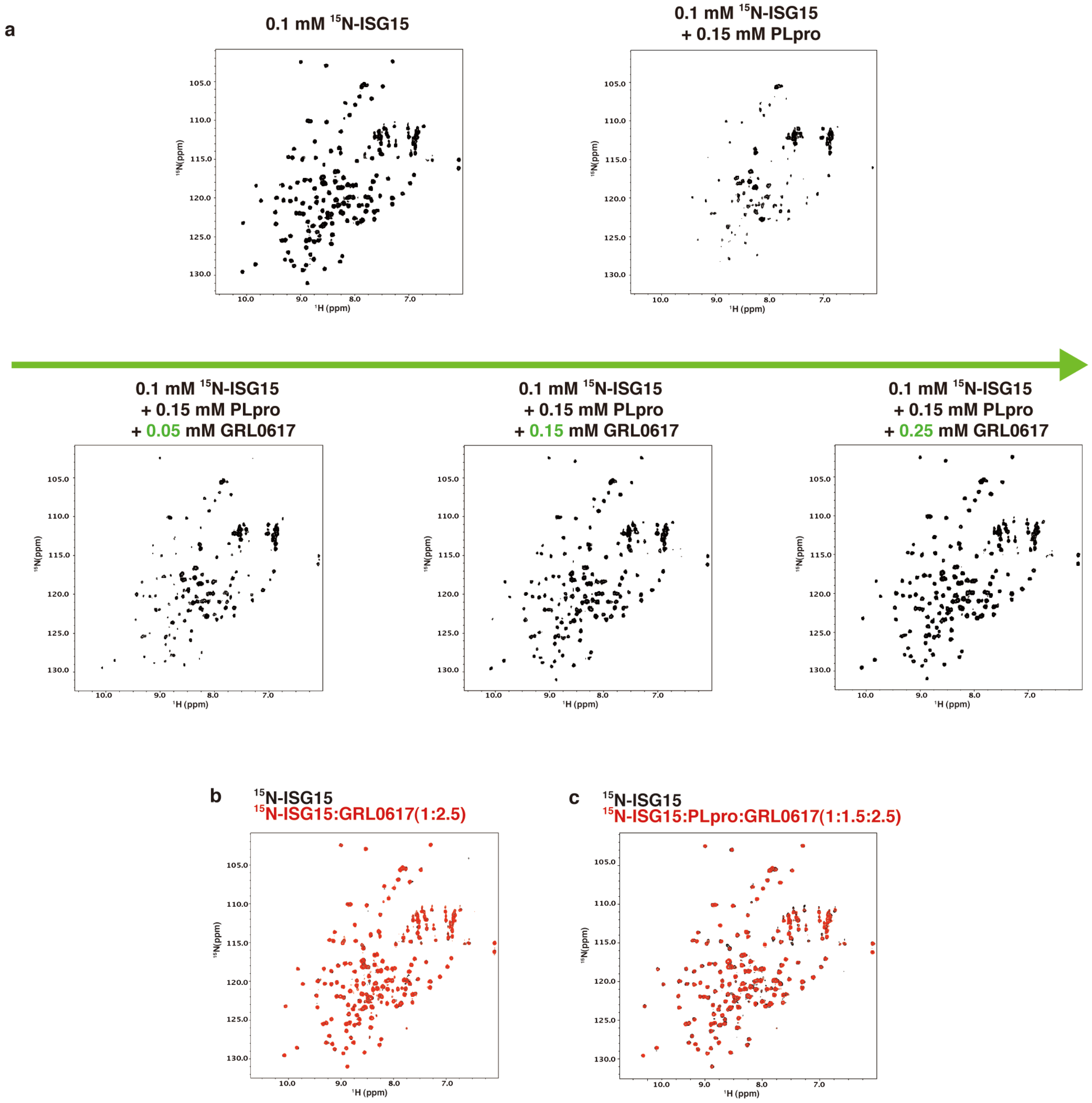

Supplementary Figure 6: GRL0617 blocks the binding of ISG15 to PLpro in a dose dependent manner.

a Addition of PLpro caused peak disappearing of ^{15}N-ISG15 due to binding. With increasing concentrations of GRL0617, the cross-peaks of ^{15}N-ISG15 gradually recovered. **b** Superposition of ^{1}H, ^{15}N-HSQC spectra at the indicated molar ratios for ^{15}N-ISG15 (black) versus the mixture of ^{15}N-ISG15 and GRL0617 (red) showing no binding of GRL0617 to ISG15. **c** Superposition

of ^1H, ^{15}N-HSQC spectra at the indicated molar ratios for ^{15}N-ISG15 (black) versus the mixture of ^{15}N-ISG15, PLpro and GRL0617 (red), showing a complete disruption of interactions between ISG15 and PLpro by GRL0617.

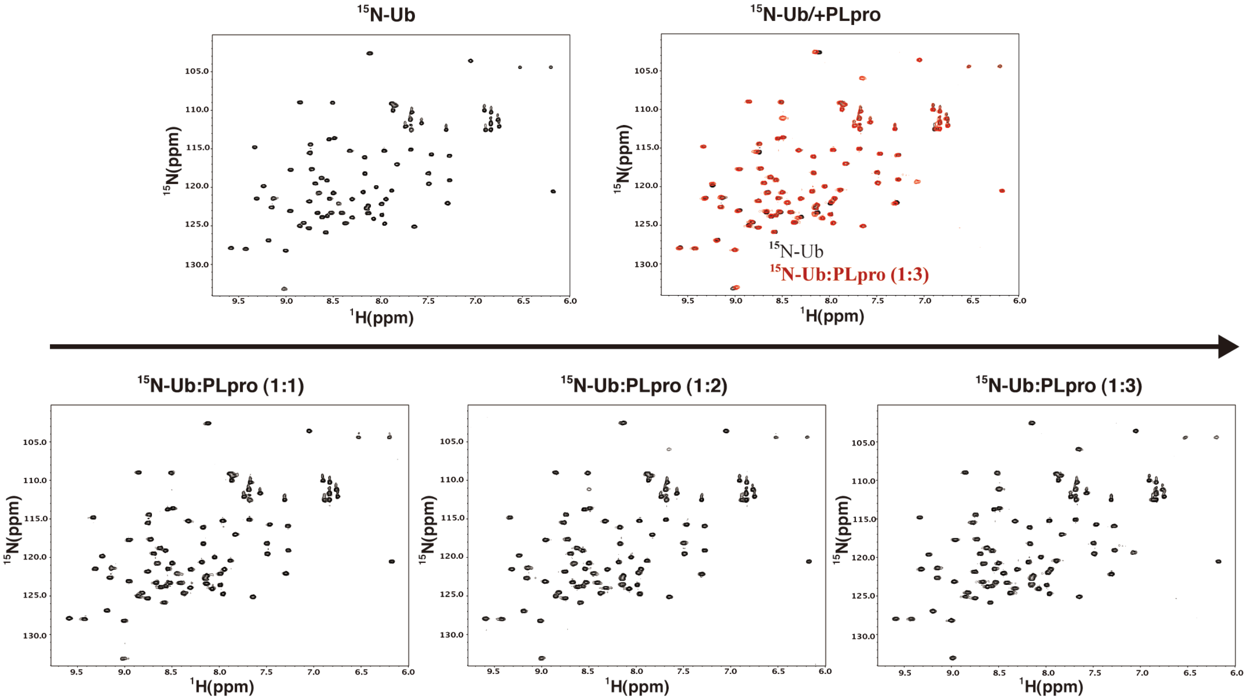

Supplementary Figure 7: No or very weak interactions between Ub and SARS-CoV-2 PLpro. ^{1}H, ^{15}N-HSQC spectra at the indicated molar ratios for ^{15}N-Ub (black) mixed with SARS-CoV-2 PLpro. Marginal spectral perturbation at 1: 3 molar ratio suggests very weak binding between monoUb and PLpro under *in vitro* conditions. The concentration of ^{15}N-Ub was 0.1 mM.

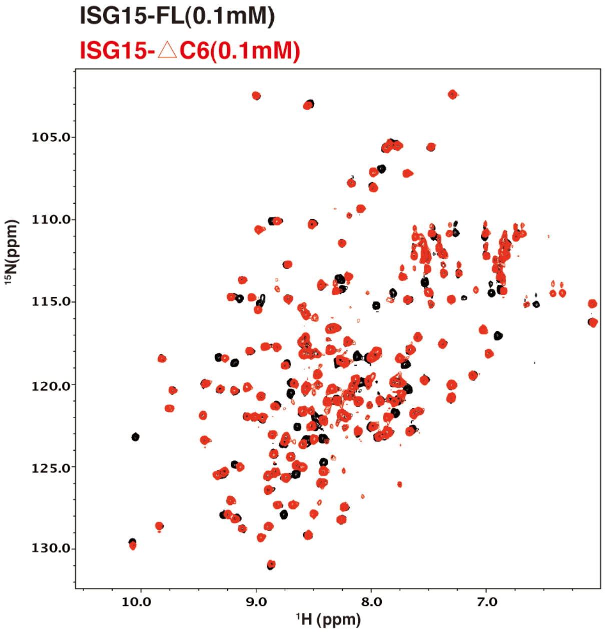

Supplementary Figure 8: The superposition of ^{1}H, ^{15}N-HSQC spectra at the indicated molar concentrations for ^{15}N-ISG15-FL (black) versus ^{15}N-ISG15-ΔC6 indicating these two proteins have basically the same fold except for the missing LRLRGG tail.

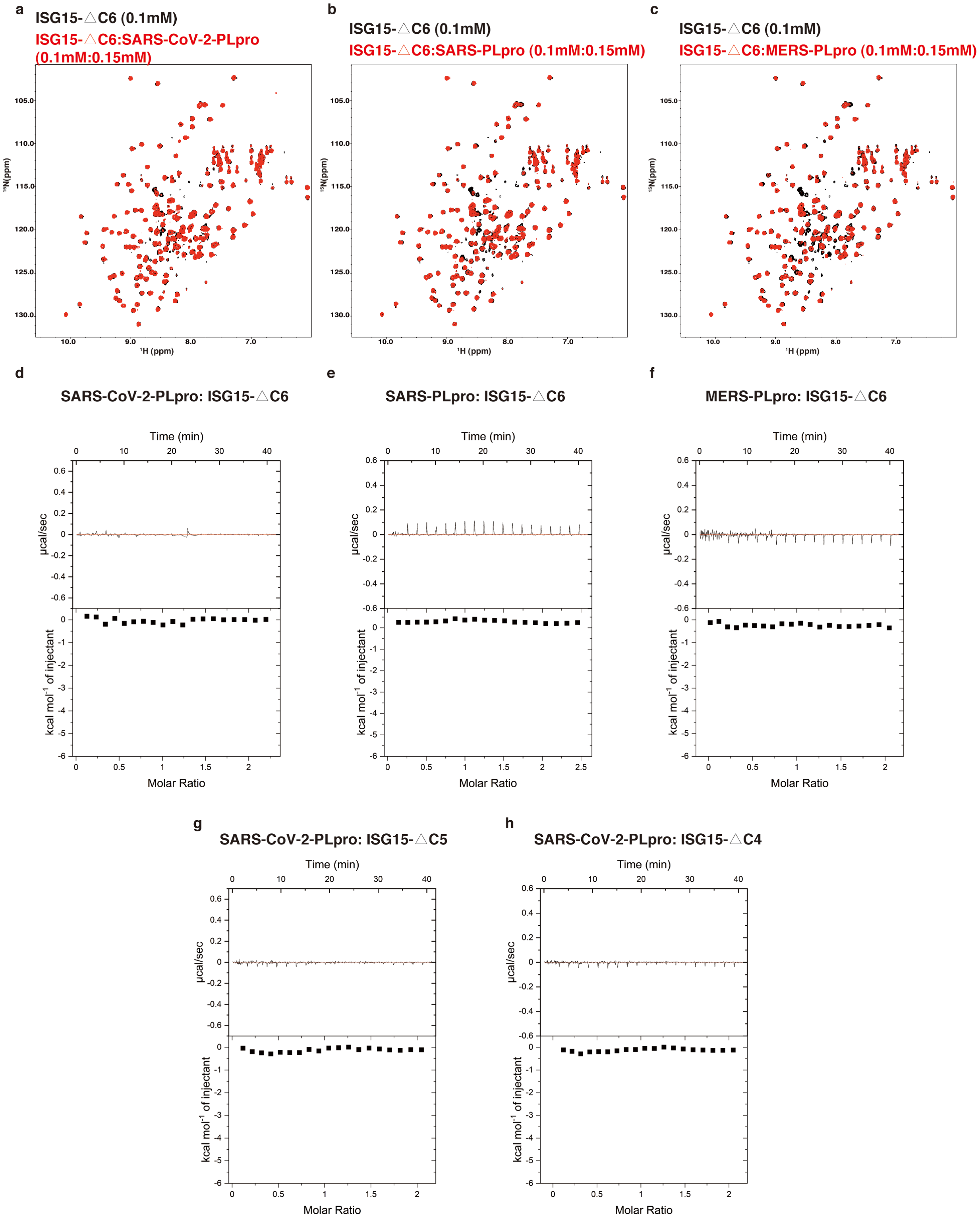

Supplementary Figure 9: Lack of interactions between ISG15 C-terminal truncated mutants with SARS-CoV-2/SARS-CoV/MERS PLpro proteins revealed by NMR and ITC.

a Superposition of ^{1}H, ^{15}N-HSQC spectra for ^{15}N-ISG15-△C6 (black) versus ^{15}N-ISG15-△C6: SARS-CoV-2 PLpro (red) indicates no or very weak interactions. **b-c** NMR indicates no or very weak interactions between ISG15-△C6 (black) and SARS or MERS PLpro (red). **d-f** ITC results showed no binding between ISG15-△C6 and SARS-CoV-2, SARS-CoV or MERS PLpro. **g** and h, ITC result showed no binding between ISG15-△C5 or ISG15-△C4 with SARS-CoV-2 PLpro.

a

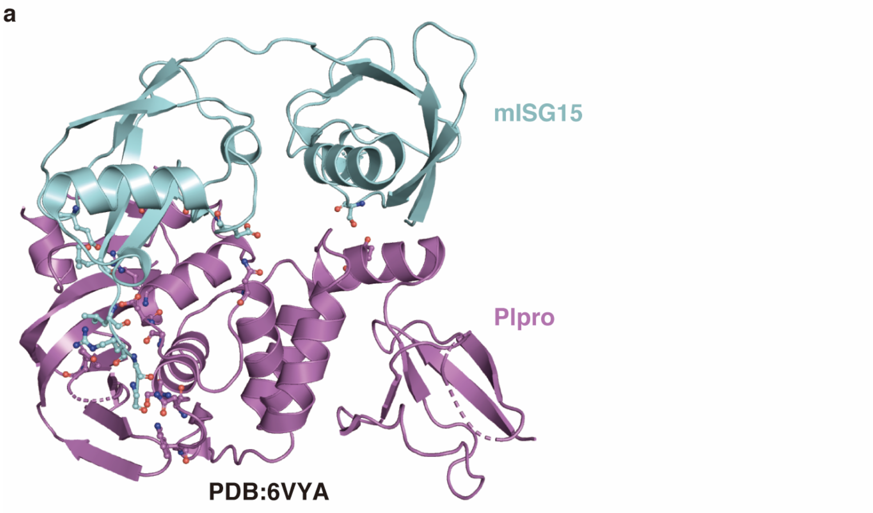

b

Hydrogen bonds XML

##	Structure 1	Dist. [Å]	Structure 2
1	C:SER 22[OG]	2.64	A:GLU 70[OE2]
2	C:GLY 126[N]	3.45	A:GLU 203[OE2]
3	C:LEU 152[N]	3.85	A:ASP 164[OD2]
4	C:ARG 153[NH1]	3.31	A:TYR 264[OH]
5	C:GLY 154[N]	2.88	A:GLY 163[O]
6	C:GLU 87[OE2]	2.57	A:ARG 166[NH1]
7	C:GLU 130[OE1]	3.83	A:ASN 156[ND2]
8	C:LEU 150[O]	3.20	A:ARG 166[NH2]
9	C:ARG 153[O]	2.62	A:TYR 264[OH]
10	C:GLY 154[O]	3.77	A:ASN 109[ND2]
11	C:GLY 155[O]	2.49	A:TRP 106[NE1]
12	C:GLY 155[O]	2.30	A:SER 111[OG]
	mISG15		PLpro

Salt bridges XML

##	Structure 1	Dist. [Å]	Structure 2
1	C:GLU 87[OE1]	3.88	A:ARG 166[NH2]
2	C:GLU 87[OE2]	2.57	A:ARG 166[NH1]
3	C:GLU 87[OE2]	2.70	A:ARG 166[NH2]
	mISG15		PLpro

6VYA

c

Hydrogen bonds XML

##	Structure 1	Dist. [Å]	Structure 2
1	D:ARG 153[NE]	2.91	C:GLU 167[OE1]
2	D:ARG 153[NH2]	2.70	C:GLU 167[OE2]
3	D:LEU 154[N]	2.75	C:ASP 164[OD2]
4	D:ARG 155[N]	3.40	C:TYR 264[OH]
5	D:ARG 155[N]	3.31	C:TYR 268[O]
6	D:GLY 156[N]	2.81	C:GLY 163[O]
7	D:GLY 128[O]	3.43	C:SER 170[OG]
8	D:ASN 151[OD1]	3.40	C:ARG 166[NH1]
9	D:LEU 152[O]	3.45	C:ARG 166[NH2]
10	D:ARG 155[O]	2.86	C:GLY 271[N]
11	D:GLY 156[O]	2.94	C:GLY 163[N]
	hCISG15		PLpro

Salt bridges XML

##	Structure 1	Dist. [Å]	Structure 2
1	D:ARG 153[NE]	2.91	C:GLU 167[OE1]
2	D:ARG 153[NE]	3.50	C:GLU 167[OE2]
3	D:ARG 153[NH2]	3.67	C:GLU 167[OE1]
4	D:ARG 153[NH2]	2.70	C:GLU 167[OE2]
	hCISG15		PLpro

6XA9

Supplementary Figure 10: Structural analysis revealed the C-terminus of ISG15 dominates its binding with SARS-CoV-2 PLpro

a the complex structure of full length mouse ISG15 and SARS-CoV-2 PLpro (PDB 6VYA [10.2210/pdb6vya/pdb]) with inter-molecular interactions illustrated. **b** PISA[3] analysis of the inter-molecular interactions between mISG15-FL and PLpro. **c** PISA analysis of the inter-molecular

interactions between human ISG15 C-UBL domain and PLpro (PDB 6XA9 [10.2210/pdb6xa9/pdb]), a large part of the binding was contributed by the C-terminus of ISG15.

Supplementary References:

1. Robert, X. & Gouet, P. Deciphering key features in protein structures with the new ENDscript server. *Nucleic Acids Res* **42**, W320-4 (2014).
2. Madeira, F. et al. The EMBL-EBI search and sequence analysis tools APIs in 2019. *Nucleic Acids Res* **47**, W636-W641 (2019).
3. Krissinel, E. & Henrick, K. Inference of macromolecular assemblies from crystalline state. *J Mol Biol* **372**, 774-97 (2007).
