# Supplementary material for: The complex structure of GRL0617 and SARS-CoV-2 PLpro reveals a hot spot for antiviral drug discovery
Source: Nat Commun. 2021 Jan 20;12:488. doi: 10.1038/s41467-020-20718-8 (PMC7817691; doi:10.1038/s41467-020-20718-8)
Supplement: Supplementary file 2 — Reporting Summary [file 41467_2020_20718_MOESM2_ESM.pdf]

nature research

Corresponding author(s): Hao Huang, Guoliang Zhang;

Last updated by author(s): Dec 11, 2020

Reporting Summary

Nature Research wishes to improve the reproducibility of the work that we publish. This form provides structure for consistency and transparency in reporting. For further information on Nature Research policies, see our Editorial Policies and the Editorial Policy Checklist.

Statistics

For all statistical analyses, confirm that the following items are present in the figure legend, table legend, main text, or Methods section.

n/a	Confirmed	
☐	☒	The exact sample size (n) for each experimental group/condition, given as a discrete number and unit of measurement
☐	☒	A statement on whether measurements were taken from distinct samples or whether the same sample was measured repeatedly
☒	☐	The statistical test(s) used AND whether they are one- or two-sided<br>*Only common tests should be described solely by name; describe more complex techniques in the Methods section.*
☒	☐	A description of all covariates tested
☒	☐	A description of any assumptions or corrections, such as tests of normality and adjustment for multiple comparisons
☐	☒	A full description of the statistical parameters including central tendency (e.g. means) or other basic estimates (e.g. regression coefficient) AND variation (e.g. standard deviation) or associated estimates of uncertainty (e.g. confidence intervals)
☒	☐	For null hypothesis testing, the test statistic (e.g. F, t, r) with confidence intervals, effect sizes, degrees of freedom and P value noted<br>*Give P values as exact values whenever suitable.*
☒	☐	For Bayesian analysis, information on the choice of priors and Markov chain Monte Carlo settings
☒	☐	For hierarchical and complex designs, identification of the appropriate level for tests and full reporting of outcomes
☒	☐	Estimates of effect sizes (e.g. Cohen's d, Pearson's r), indicating how they were calculated

Our web collection on statistics for biologists contains articles on many of the points above.

Software and code

Policy information about availability of computer code

Data collection	CrysAlisPro(41_64.64a);Image lab software ver (3.0);Bruker advance III HD 600MHz
Data analysis	CrysAlisPro(41_64.64a);CCP4 (v7.0.066); Coot (v0.8.9.1);Phenix(1.18.2_3874);Pymol(2.3.5);Image lab software ver (3.0);GraphPad Prism (v6.0.4);NMRView(5.2);Microcal Origin (5.0);Microcal-ITC200 (1.26.4)

For manuscripts utilizing custom algorithms or software that are central to the research but not yet described in published literature, software must be made available to editors and reviewers. We strongly encourage code deposition in a community repository (e.g. GitHub). See the Nature Research guidelines for submitting code & software for further information.

Data

Policy information about availability of data

All manuscripts must include a data availability statement. This statement should provide the following information, where applicable:

- Accession codes, unique identifiers, or web links for publicly available datasets
- A list of figures that have associated raw data
- A description of any restrictions on data availability

Coordinates and structure factors were deposited in the PDB under accession code 7CJM [10.2210/pdb7cjm/pdb] (GRL0617 bound PLproC111S). Protein sequence for SARS-CoV-2 PLpro (amino acids, 746-1060) of Nsp3 protein from SARS-CoV-2 (Nsp3; YP_009742610.1). Protein sequence for SARS PLpro (amino acids, 723-1037) of Nsp3 protein from SARS (Nsp3; NP_828862.2). Protein sequence for MERS PLpro (amino acids, 627-950) of Nsp3 protein from MERS (Nsp3; YP_009047231.1). Protein sequence for Homo sapiens ISG15 (amino acids, 1-157) (ISG15; NP_005092.1). Protein sequence for Mus musculus USP18(amino acids, 46-368) (USP18; CAJ18436.1P). All relevant data are available upon request. Source data are provided with this paper.

Field-specific reporting

Please select the one below that is the best fit for your research. If you are not sure, read the appropriate sections before making your selection.

[x] Life sciences [ ] Behavioural & social sciences [ ] Ecological, evolutionary & environmental sciences

For a reference copy of the document with all sections, see nature.com/documents/nr-reporting-summary-flat.pdf

Life sciences study design

All studies must disclose on these points even when the disclosure is negative.

Sample size	Experiments were repeated three times with similar results and sample size was chosen based on the consistency and significance a of measured differences between groups. No sample size calculation was done.
Data exclusions	No data were excluded from analysis.
Replication	We have repeated each experiment in the manuscript at least three times to ensure consistent results. At least three successful attempts.
Randomization	No randomization was necessary as various infected cells were recorded and analyzed by a computer program.
Blinding	Blinding was not relevant for the experiments done as various infected cells were analyzed by a computer program.

Reporting for specific materials, systems and methods

We require information from authors about some types of materials, experimental systems and methods used in many studies. Here, indicate whether each material, system or method listed is relevant to your study. If you are not sure if a list item applies to your research, read the appropriate section before selecting a response.

Materials & experimental systems

n/a	Involved in the study	
[ ]	[x]	Antibodies
[ ]	[x]	Eukaryotic cell lines
[x]	[ ]	Palaeontology and archaeology
[x]	[ ]	Animals and other organisms
[x]	[ ]	Human research participants
[x]	[ ]	Clinical data
[x]	[ ]	Dual use research of concern

Methods

n/a	Involved in the study	
[x]	[ ]	ChIP-seq
[x]	[ ]	Flow cytometry
[x]	[ ]	MRI-based neuroimaging

Antibodies

Antibodies used

Ubiquitin (Cat#3936, Cell Signaling Technology, 1:1000), ISG15 (Cat#703131, Thermo Fisher Scientific, 1:1000), GFP (Cat#AE012, Abclonal, 1:1000), GAPDH (Cat#AC002 Abclonal, 1:5000), anti-rabbit-HRP (Cat#ADI-SAB-300-J, Enzo Life Sciences, 1:10000) anti-mouse-HRP (Cat#sc-516102, Santa Cruz Biotechnology, 1:10000)

Validation

Ubiquitin (Cat# 3936, Provider: Cell signaling Technology)
Validation statement from the manufacturer: Ubiquitin (P4D1) Mouse mAb detects ubiquitin, polyubiquitin and ubiquitinated proteins. This antibody may cross-react with recombinant NEDD8.
Validation found at provider's website: https://www.cellsignal.com/products/primary-antibodies/ubiquitin-p4d1-mousemab/3936

ISG15 (Cat# 703131, Thermo Fisher Scientific)
Validation statement from the manufacturer: ISG15 Recombinant Rabbit Monoclonal Antibody; This Antibody was verified by Knockout to ensure that the antibody binds to the antigen stated.
Validation found at provider's website: https://www.thermofisher.com/cn/zh/antibody/product/ISG15-Antibody-clone-7H29L24-Recombinant-Monoclonal/703131

GFP (Cat#AE012, Abclonal)
Validation statement from the manufacturer: A synthetic peptide corresponding to a sequence within amino acids 1-100 to the N-terminus of GFP protein. Western blot analysis of over-expressed GFP fusion protein in 293T cell using GFP-Tag antibody (AE012).
Species Reactivity: All Species Expected
Validation found at provider's website: https://www.abclonal.com.cn/catalog/AE012

GAPDH (Cat#AC002)

Validation statement from the manufacturer: Recombinant of human GAPDH; Western blot analysis of extracts of various cell lines, using GAPDH antibody.
Species Reactivity: Human, Mouse, Rat
Validation found at provider's website: https://www.abclonal.com.cn/catalog/AC002

Anti-rabbit-HRP (Cat#ADI-SAB-300-J)
Validation statement from the manufacturer: Based on immunoelectrophoresis and/or ELISA, the antibody reacts with whole molecule rabbit IgG. It also reacts with the light chains of other rabbit immunoglobulins. No antibody was detected against non-immunoglobulin serum proteins.
Species Reactivity: Rabbit
Validation found at provider's website: https://www.enzolifesciences.com/ADI-SAB-300/goat-anti-rabbit-igg-polyclonal-antibody-hrp-conjugate/

Anti-mouse-HRP (Cat#sc-516102)
Validation statement from the manufacturer: Mouse IgGκ light chain binding protein (m-IgGκ BP) conjugated to horseradish peroxidase (HRP) is a strongly recommended alternative to conventional antimouse IgG secondary antibodies for Western Blotting (WB) and immunohistochemical (IHC) signal enhancement.
Species Reactivity: mouse
Validation found at provider's website: https://www.scbt.com/p/m-igg-kappa-bp-hrp?requestFrom=search

Eukaryotic cell lines

Policy information about cell lines

Cell line source(s)	HEK293T (ATCC);Vero E6 (Shanghai Institutes for Biological Sciences, Chinese Academy of Sciences); sf9 (Thermo Fisher Scientific)
Authentication	All cells were with authentication. The authentication was performed by morphology check under microscopes and growth curve analysis.
Mycoplasma contamination	We confirm that all cells were mycoplasma negative.
Commonly misidentified lines (See ICLAC register)	No commonly misidentified cell lines were used.
